# Meta-evolutionary exome analysis identifies novel type 2 diabetes mellitus genes in the UK Biobank and All of Us

**DOI:** 10.1371/journal.pgen.1011889

**Published:** 2025-09-30

**Authors:** Kevin Wilhelm, Jennifer Asmussen, Kwanghyuk Lee, Maryam Samieinasab, Emmanuel Asante, Marek Kimmel, Olivier Lichtarge

**Affiliations:** 1 Department of Molecular and Human Genetics, Baylor College of MedicineHouston, Texas, United States of America; 2 Department of Statistics, Rice University, Houston, Texas, United States of America; 3 Department of Biochemistry and Molecular PharmacologyHouston, Texas, United States of America; 4 Computational and Integrative Biomedical Research Center, Baylor College of MedicineHouston, Texas, United States of America; University of Melbourne, AUSTRALIA

## Abstract

Type 2 diabetes mellitus (T2DM) risk is heavily influenced by genetics, yet current association tests have explained only parts of its heritability. We developed MEVA (Meta-Evolutionary Action), a meta-analytic framework that integrates three complementary methods—EAML, Sigma-Diff, and GeneEMBED—to assess the functional burden of protein-coding variants using evolutionary data. MEVA was applied to exome data from 28,115 T2DM cases and 28,115 controls in the UK Biobank (UKB), identifying 101 genes (p < 1e-5). MEVA outperformed its component methods, each of which substantially outperformed a conventional burden test (MAGMA), in recovering known T2DM genes (AUROC = 0.925) and maintaining robustness in progressively smaller cohorts (AUROC = 0.917). MEVA showed significant enrichment for T2DM-related loci (p = 6.8e-10, p = 2.0e-34), protein interactions (z = 4.6, z = 4.2), pathways (p = 1.3e-6, z = 2.0), phenotypes (p = 1.3e-21, z = 9.1), and literature mentions (z = 7.2). Replication in 16,915 T2DM cases and 16,915 controls from All of Us (AoU) yielded 99 genes (p < 1e-5), 23 of which were also recovered in the UKB cohort – far exceeding random chance. These included established genes (*SLC30A8, WFS1, HNF1A*) and less-characterized candidates (*NRIP1, ADAM30, CALCOCO2, TUBB1, ZFP36L2, WDR90*). Notably, *NRIP1* loss-of-function variants were associated with increased T2DM risk in both the UKB (OR = 1.09, FDR = 5.4e-4) and AoU (OR = 1.09, FDR = 0.046), and *TUBB1* and *CALCOCO2* gain-of-function variants showed consistent risk effects (FDR < 0.05). Pathway analyses revealed convergence on endoplasmic reticulum chaperone complexes (FDR = 0.02) and Hippo signaling (FDR = 8.5e-4). Finally, all 177 candidate genes were functionally prioritized using ten orthogonal criteria to guide experimental follow-up. These results demonstrate that combining complementary, impact-aware association tests increases sensitivity, improves replication, and expands the catalog of genetic risk factors for T2DM.

## Author summary

Many genetic risk factors for type 2 diabetes mellitus (T2DM), a major global health concern, remain unknown. Genetic studies have found hundreds of T2DM risk regions but face two key challenges. First, most findings are non-coding and regulatory, making it hard to identify the associated gene(s). Second, studies of protein-altering variants often treat every change as equally damaging. To address the challenge of assessing variant damage, our tool, MEVA, pinpoints genes by weighing variants with their predicted functional impact. For every protein-altering variation, MEVA asks, “How much will it hinder function?”, merging deleterious estimates from complementary scores for each gene. We applied MEVA to nearly 60,000 British patients and, separately, to more than 30,000 U.S. patients, identifying 177 genes altogether. Of these, 23 genes replicated in both populations, and a combined analysis of the cohort-specific genes revealed enrichment in shared diabetes-related pathways, such as the stress response in insulin-producing cells. Using independent functional evidence, these genes were prioritized for follow-up experiments and potential therapeutic studies. Our findings show that: 1) weighing the predicted deleterious impact of gene variations improves disease-specific gene discovery, 2) integrating approaches that use this score outperforms each single method, and 3) this strategy uncovers novel biologically-relevant genetic risk factors to enrich diabetes genetics.

## Introduction

The lifetime risk of type 2 diabetes mellitus (T2DM) approaches 40% in the U.S., and by 2045, it is projected to affect 700 million people worldwide [1]. T2DM is characterized by hyperglycemia, typically indicated by elevated glycated hemoglobin (HbA1c), resulting from impaired insulin secretion amid progressive insulin resistance. Over time, micro- and macrovascular damage leads to diverse complications including blindness, kidney failure, and cognitive impairment [2,3]. Risk arises not only from lifestyle factors, such as body mass index (BMI), but also from genetics [4], with heritability estimates ranging from 30% to 70% [5]. Genome-wide association studies (GWAS), which primarily detect common variants without *in silico* estimates of a variant’s impact (“impact-agnostic”), have revealed a polygenic architecture [6], identifying over 700 loci [7] that influence metabolic, immune, and hormonal pathways across the pancreas, liver, adipose tissue, and skeletal muscle [8]. While most signals fall within non-coding regions and are presumed regulatory [9], partitioned-heritability analyses attribute over 50% of T2DM risk to these non-coding variants, yet the ~ 2% of the genome that is protein-coding contributes an outsized 14–24% [10,11]. Several loci map to well-established genes, such as *KCNJ11*, *ABCC8, SLC30A8,* and *PPARG*, which regulate glucose sensing, insulin secretion, and adipose tissue function [12]. More recently, rare-variant exome-sequencing studies have nominated new candidate genes, such as *GIGYF1* [13,14], *MAP3K15* [15,16] and *FAM234A* [16]. Yet, both common and rare variant approaches explain only a fraction of T2DM heritability [17,18], in part because they sample a limited set of allele frequencies, functional impacts, or ancestries, which can limit their power to unmask other risk genes [19–21].

To address these limitations, we developed MEVA, a method that integrates three gene-level association tests that account for the functional impact of coding variants (“impact-aware”). MEVA begins by estimating the likely effect of each protein-coding variant using Evolutionary Action (EA), a score derived from evolutionary history. EA is calculated as the product of the phylogenetic importance of the mutated residue [22] and the magnitude of the amino acid substitution [23]. In blinded variant annotation challenges, EA has consistently ranked among the top-performing methods [24,25]. By comparing the aggregate EA burden between cases and controls, we can identify genes significantly associated with disease risk, as demonstrated previously in cancer [26,27], early-onset coronary artery disease [28], autism [29], and Alzheimer’s disease [30–32].

Here, we focus on three complementary approaches that each leverage EA scores to detect gene-level associations. The first is EAML, an ensemble machine learning model that evaluates each gene independently [28,31]. The second, Sigma-Diff, quantifies evolutionary selection pressure using EA burden between cases and controls. The third, GeneEMBED, interprets differences in EA burden in the context of protein–protein interaction networks [32]. Although these methods differ in design, they each aim to identify genes with an abnormal variant impact distribution. To combine their strengths, we applied the Cauchy combination test (CCT) [33], a statistically rigorous approach that retains power even when input tests are correlated—a strategy validated in multiple prior genomic studies [34,35].

To evaluate MEVA, we applied it to 28,115 T2DM cases and matched controls from the UK Biobank (UKB) [36], assessed the resulting gene associations using functional validation metrics, and then tested replication in an independent cohort of 16,915 T2DM cases and controls from All of Us (AoU) [37]. This analysis replicated 23 genes, revealed convergence on shared diabetes-related pathways, and yielded a total of 177 candidate genes that we prioritized for follow-up studies, including several (*NRIP1, CALCOCO2, WDR90, TUBB1, ZFP36L2, ADAM30, PFKM, H6PD, NUAK2, UBAP2*) that ranked highly despite limited prior association with diabetes in the literature.

## Results

### MEVA improves gene discovery to identify 101 high-confidence candidates

**Fig 1:**
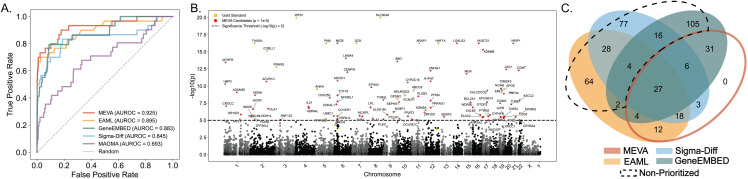
MEVA improves gold standard prioritization and prioritizes 101 candidate genes. (A) AUROC curves for recovery of 31 gold standard genes by MEVA, its component methods, and MAGMA. (B) Manhattan plot of MEVA results (-log10 p-value vs. chromosome; each dot represents a gene). MEVA candidates (n = 101; p < 1e-5) are shown in red, gold standard genes in yellow, and all other genes in gray/black. (C) Venn diagram showing the overlap between MEVA and its component methods. Non-prioritized genes, those that were significant in at least one component method but not in MEVA, are outlined in dotted black.

To identify genes associated with T2DM, we analyzed UKB exomes and, after applying phenotypic (S1 Table) and sequencing quality control (QC) filters, assembled 28,115 T2DM cases and 28,115 unaffected, healthy controls (Methods). Table 1 summarizes demographic, anthropometric, and laboratory measurements, confirming expected differences in age, BMI, HbA1c, and glucose (all *p* < 0.0001), while matching for sex (60.6% male in both groups). Notably, T2DM cases had lower LDL cholesterol, which was largely explained by a higher prevalence of statin use (OR = 4.9, p < 0.001). We scored protein-coding variants (allele frequency (AF) < 50%) with EA and analyzed them with Multi-marker Analysis of GenoMic Annotation (MAGMA) [38], a benchmark impact-agnostic method, and with the three EA-based methods: EAML, GeneEMBED, and Sigma-Diff. We then merged the three EA-based method p-values with the Cauchy combination test (CCT) to create MEVA’s overall association statistic. Initial evaluation of each method’s ability to rank, by p-value, 31 gold standard T2DM genes (S2 Table) defined by independent genetic and experimental evidence from the Open Targets Platform [39], showed that MEVA achieved the best ranking (AUROC = 0.925, Fig 1A), outperforming EAML (0.90), GeneEMBED (0.88), Sigma-Diff (0.85) and MAGMA (0.69, S3 Table). While MEVA’s improvement over EAML is modest, it was statistically significant (DeLong p = 0.008) and further supported by the capture of 21 gold standards in the top 200 genes, compared to 20 for EAML. To further compare performance across methods, we defined high-confidence gene sets using method-specific significance thresholds. Applying the strictest cut-off (FDR < 1.7e-3, p < 1e-5), MEVA identified 101 genes (Fig 1B), while EAML found 159 genes with FDR < 0.1, GeneEMBED had 195 with FDR < 0.1, Sigma-Diff had 179 with |*z*| ≥ 3 (FDR < 0.266), and MAGMA yielded 176 genes (*p* < 0.01, FDR < 0.922). We set the MAGMA threshold to match suggestive thresholds used in similar gene-based tests [40,41] and to yield a candidate list of comparable size, ensuring fair downstream comparisons. Every MEVA gene was significant in at least one EA method, whereas 296 genes that met the selection threshold in the individual EA methods fell below MEVA’s threshold and were considered “non-prioritized” by that method (Fig 1C). MEVA prioritized 95 genes that MAGMA failed to identify; the two methods overlapped by only six genes (*GCK, HNF4A, PAM, KCNJ11*, *TCF19, MTMR3*). One example is *CALCOCO2* (MEVA *p* = 4.6e-10; MAGMA *p* = 0.81), a gene newly implicated in insulin secretion [42]. Together, these results suggest that MEVA integrates complementary impact-aware tests to improve the prioritization of T2DM genes and reveal candidate genes overlooked by conventional methods.

**Table 1:** Clinical and demographic characteristics of UKB T2DM cases (n = 28,115) compared with controls (n = 28,115). Values are shown as mean ± standard deviation or percentage. P-values were determined by a two-sample t-test or Fisher’s exact test (Male, %). “NA” indicates data were not applicable or unavailable for controls. Anthropometry and Laboratory Measurements were taken at patient enrollment.

Characteristic	T2DM Cases (n = 28,115)	Controls (n = 28,115)	P-Value
**Demographics**			
Age (y), Mean ± SD	77.16 ± 6.87	77.71 ± 4.54	<0.0001
Male, %	60.61%	60.61%	1.0
Age at Diagnosis (y), Mean ± SD	65.34 ± 8.20	NA	NA
**Anthropometry**			
BMI (kg/m^2^), Mean ± SD	31.75 ± 5.66	30.20 ± 4.04	<0.0001
Body Fat %, Mean ± SD	34.54 ± 8.39	33.28 ± 8.24	<0.0001
Whole Body Fat Mass (kg), Mean ± SD	31.88 ± 11.38	29.08 ± 8.83	<0.0001
**Laboratory Measurements**			
HbA1c (%), Mean ± SD	6.4 ± 1.1	5.37 ± 0.34	<0.0001
Glucose (mmol/mol), Mean ± SD	6.52 ± 2.64	4.98 ± 0.61	<0.0001
Total Cholesterol (mmol/L), Mean ± SD	5.04 ± 1.26	5.82 ± 1.12	<0.0001
Low-Density Lipoprotein (mmol/L), Mean ± SD	3.12 ± 0.94	3.69 ± 0.85	<0.0001
High-Density Lipoprotein (mmol/L), Mean ± SD	1.20 ± 0.31	1.38 ± 0.34	<0.0001

### MEVA prioritizes T2DM-relevant genes more effectively than MAGMA and its component methods

**Fig 2:**
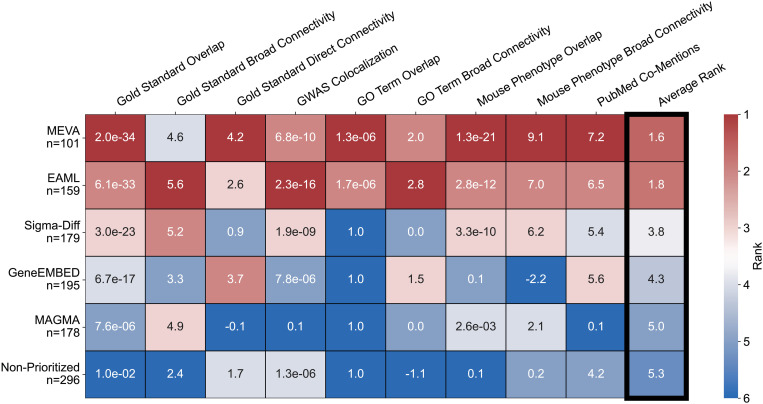
MEVA outperforms its component methods, non-prioritized genes, and MAGMA in T2DM-related genetic, pathway, phenotype, and literature enrichments. Heatmap displaying method performance across multiple validation criteria for genes significant in MEVA, the component methods, non-prioritized genes, and MAGMA. Cells are color-coded by rank (red = best, blue = worst) and annotated with either p-values (GWAS Colocalization, Gold Standard, GO Term, and Mouse Phenotype Overlap) or z-scores (Gold Standard Broad and Direct Connectivity, GO Term and Mouse Phenotype Broad Connectivity, and 1 + PubMed Co-Mentions).

To further evaluate performance and assess the relevance of novel candidate genes, we benchmarked each algorithm’s significant gene set against nine independent criteria spanning T2DM genetics, pathways, phenotypes, and literature (Fig 2). MEVA performed the best across all metrics, ranking first in six tests, second in two, and fourth in one (mean rank = 1.6). It also retained the top position after excluding T2DM gold standard genes. EAML was the next best performing method, ranking first in three tests, second in five, and third in one (mean = 1.8). MAGMA and the 296 non-prioritized genes scored the lowest across nearly all criteria. These results place MEVA as the top-performing method overall.

Starting with the subset of the nine criteria related to T2DM genetics, we first tested each method’s overlap with the 31 gold standard T2DM genes. MEVA recovered 18 gold standard genes, achieving the strongest enrichment of any method (p = 2.0e-34; S1A Fig). This performance was slightly better than EAML (p = 6.1e-33) and represented a 13 gene improvement over MAGMA. Next, we tested first-neighbor protein interaction links to the gold standards in the STRING v12 network [43]. MEVA showed the strongest enrichment (8 direct links; *z* = 4.2; S1B Fig), outperforming GeneEMBED’s 15 links (*z* = 3.7) and MAGMA’s three links. When we extended our network analysis to include second-order and beyond connections via network diffusion [44], MEVA showed significant relatedness to the gold standards (AUROC = 0.74; *z* = 4.6; S1C Fig), but trailed EAML (*z* = 5.6) and, marginally, MAGMA (*z* = 4.9). Detailed results for all of the methods are provided in S1D-R Fig. Finally, we tested each gene set for genomic colocalization (0.5 Mbp window) with an expanded true positive set of 4,005 genome-wide significant T2DM GWAS loci from the GWAS Catalog [9]. MEVA performed second-best, finding 77 of 101 (76%) genes near known signals, 25 percentage points above MAGMA (51%, p = 0.1) and only two points below EAML (78%). Notably, 58 of the 77 colocalized MEVA genes are different from those currently annotated in GWAS Catalog, suggesting that alternative causative genes may underlie the reported association.

We next examined whether candidate gene sets were enriched in T2DM-related pathways by testing for enrichment using Gene Ontology-Biological Process (GO-BP) terms [45,46]. MEVA candidates were enriched for 51 GO-BP terms (FDR ≤0.05, S2A Fig and S4 Table). Against curated pathways from the Type 2 Diabetes Knowledge Portal [47] (T2DKP), MEVA showed the greatest overlap, sharing four pathways (S2B Fig and S5 Table) and outperforming EAML’s three pathways, while MAGMA identified no significant pathways. MEVA overlaps with T2DKP included *pancreas development* and *regulation of insulin secretion*. To assess broad pathway similarity to the curated T2DKP terms, we also performed a graph-based diffusion comparison using the GO-BP ontology structure, excluding overlapping pathways. MEVA’s GO-BP enriched terms were significantly related to T2DKP terms (AUROC = 0.69, *z* = 2.0; S2C Fig), second only to EAML (*z* = 2.8). Top MEVA driver terms included *response to hexose* (similarity percentile = 99.8) and *homeostatic process* (98.9). Full results for the other methods are shown in S2D-K Fig.

To assess physiological relevance, we tested for the enrichment of *in vivo* phenotypes using the Mouse Genome Informatics (MGI) database [48] and compared against a curated set of 261 T2DM-associated traits [49] (S6 Table). MEVA genes were enriched for 47 mouse phenotypes (q < 0.05; S7 Table and S3A Fig), including 19 T2DM-related phenotypes (*p* = 1.3e-21; S3B Fig), surpassing EAML’s 12 terms and MAGMA’s 3 terms. MEVA’s key overlaps included *increased circulating glucose level* and *decreased insulin secretion*. In a broader phenotype graph-based diffusion analysis using the phenotype ontology, MEVA again ranked the highest (AUROC = 0.68, *z* = 9.1; S3C Fig), ahead of EAML and MAGMA. Top MEVA drivers included *increased skeletal muscle glycogen level* (*H6PD, PFKM*; similarity percentile = 93.8) and *abnormal active avoidance behavior* (*TH, PLAT*; 89). Complete phenotype enrichment results for all methods are shown in S3D-R Fig.

Finally, we assessed the enrichment of genes with existing T2DM literature evidence by querying the PubMed database for the co-mention of each gene with “Type 2 Diabetes.” MEVA performed the best with 65 genes having at least one co-mention (*z* = 7.2; S4A Fig) and 16 having more than 50 co-mentions (*z* = 14.1; S4B Fig), surpassing EAML and MAGMA, as well as the other methods (S4C-L Fig). Together, these data show that MEVA, on average, outperforms MAGMA and its component methods across multiple independent validation frameworks, highlighting its ability to identify genes strongly supported by existing genetics, pathway knowledge, phenotypes, and literature, while deprioritizing likely false-positive genes.

As MEVA and EAML were the top performing methods, we sought to dissect their unique strengths by analyzing each exclusive gene set (S5 Fig). The 98 EAML-unique genes excelled at recovering known biology, showing stronger enrichment for GWAS colocalization (p = 2.2e-6) and mouse phenotype overlap (p = 2.0e-2) while identifying one additional gold standard (*RREB1*). However, the 40 MEVA-unique genes better identified functionally related candidates, with stronger protein-interaction links to gold standards (*z* = 3.0, *z* = 4.1) and diabetes-related GO terms (*z* = 2.7), such as *response to stress*. This highlights MEVA’s strength in generating new functional hypotheses. One example is *H6PD*, which was missed by EAML (FDR = 0.91) but prioritized by MEVA (p = 4.8e-11) via its network-based component (GeneEMBED) and is supported by a compelling biological context, including its direct interaction with the gold standard *GCK* and its link to muscle glycogen phenotypes in mice [50]. These results suggest that while EAML is effective at recovering known biology, MEVA’s ensemble approach excels at identifying novel, hypothesis-generating candidates.

To test whether this performance was driven solely by known disease genes, we removed the 31 gold standards from each gene set and repeated all analyses (S6 Fig). MEVA again ranked first overall, driven by T2DM-related protein interactions (broad: *z* = 5.5; first-neighbor: *z* = 4.3) and mouse phenotypes (*z* = 2.1). EAML ranked second-best and MAGMA remained tied for last. These results demonstrate that MEVA’s advantage is not dependent on well-established genes but instead reflects its ability to prioritize overlooked candidates with independent functional support.

### MEVA remains robust and accurate in smaller cohorts

**Fig 3:**
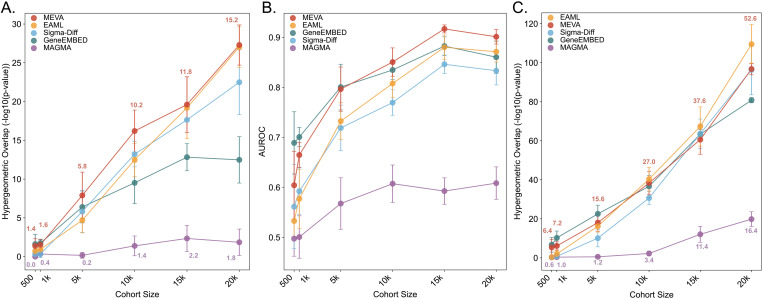
MEVA remains robust in smaller cohorts. (A) Hypergeometric overlap of the top 100 genes (ranked by p-value) with the gold standards. The mean number of overlapping genes is indicated for MEVA (red) and MAGMA (purple). Cohort size indicates the number of T2DM cases and matched health controls (e.g., 500 vs. 500 is marked as 500). (B) AUROC for recovery of gold standard genes across full p-value rankings in the down-sampled experiments. (C) Hypergeometric overlap of the top 100 genes (ranked by p-value) between the full cohort and down-sampled experiments for each method. The mean number of overlapping genes is indicated above the bars for MEVA (red) and for MAGMA (purple).

To assess the robustness of each method and inform future experimental design, we performed an empirical power analysis by evaluating progressively smaller UKB cohorts. This approach is essential for understanding how MEVA’s performance scales with sample size, a common practice for complex models where formal power calculations are intractable [51,52]. At each reduced sample size, we assessed performance based on the recovery and ranking of gold standard genes, as well as the consistency of the top-ranked gene lists. MEVA both recovered and ranked T2DM gold standards better than any other method and achieved second-best in consistency for top-ranked genes, supporting its utility in reduced sample sizes. Specifically, we randomly sampled six smaller cohorts (20k, 15k, 10k, 5k, 1k, and 500 cases with equal controls) and ran each method five times per cohort (30 runs per method). We first tested gold standard recovery by measuring the overlap between the 31 gold standard genes and the top 100 genes from each down-sampled iteration (Fig 3A and S8 Table). MEVA and EAML were the top-performing methods, with MEVA showing better recovery in the smaller sample sizes. GeneEMBED showed intermediate performance that plateaued in larger cohorts, while MAGMA showed minimal recovery at any sample size. To illustrate this performance gap, MAGMA required a cohort of 10k samples to recover, on average, the same number of gold standards that MEVA identified with only 500. Notably, MEVA’s overlap with the gold standards remained significant down to a cohort size of 2,500 cases and 2,500 controls (average p = 0.02), suggesting a potential minimum sample size for reliable gene discovery. Next, we tested rank accuracy by calculating AUROC against the 31 gold-standard genes (Fig 3B). GeneEMBED performed the best in the smallest arms, after which its performance plateaued. MEVA’s performance rose with cohort size, becoming the best-performing method in the 10k arm and peaking in the 15k arm (mean AUROC = 0.917 ± 0.008). All EA methods showed a modest dip in the 20k arm (MEVA mean AUROC = 0.901), driven by lower rankings for *TPCN2, GLP1R, AKT2, RFX6,* and *FGFR4* (S7 Fig). Finally, we measured intra-method consistency, assessing the overlap of the top 100 smaller cohort genes and the top 100 full cohort genes (Fig 3C and S8 Table). All EA-based methods were more consistent than MAGMA. EAML showed the highest consistency, with MEVA a close second. This trend is evident in the 10k cohorts, where the top 100 lists overlapped by an average of 28.2 genes in EAML (mean *p* = 1.7e-35), 27 genes in MEVA (mean *p* = 1.4e-32), and only 3.4 genes in MAGMA (mean *p* = 0.04). Notably, both MEVA and EAML captured *ARAP1, PPARG,* and *SLC30A8* in every 10k cohort run, but MEVA alone captured the less-characterized genes of interest *CALCOCO2* and *NRIP1* in two runs. Moreover, the genes that MEVA consistently ranks in the top 100 are likely T2DM-associated, including gold standard genes (*SLC30A8* in 22/30 runs; *WFS1* in 20/30; *ARAP1* in 19/30) and other less-characterized candidates (*NRIP1* in 17/30, *CALCOCO2* in 12/30; S9 Table). Together, these data show that MEVA retains T2DM specificity and robustness even when sample sizes fall by orders of magnitude, supporting its use in smaller cohorts.

### Cross-cohort replication of T2DM genes and convergence on shared pathways

Given MEVA’s robustness, we asked whether its UKB-derived predictions would replicate in an independent cohort. We applied MEVA to 16,915 non-Hispanic, white T2DM cases and matched healthy controls from AoU (Methods; S10 and S11 Tables), where demographic, anthropometric, and laboratory measures (Table 2) mirrored UKB differences when comparable data were available. Applying the same significance threshold, MEVA identified 99 genes and recovered 11 gold standards (p = 2.0e-18), consistent with down-sampling expectations for this sample size. To benchmark against a non-coding-inclusive method, we applied MAGMA to AoU WGS, including coding, intronic, and ±10kb flanking variants as recommended [38]. At p < 0.01, MAGMA identified 58 genes; none overlapped the gold standards, and the set lacked T2DM signal by GWAS colocalization, GO pathways, mouse phenotypes, and PubMed. Across AoU, MEVA remained the top method, supported by gold-standard recovery, GWAS colocalization (73%; p = 1.1e-5), and literature co-mentions (*z* = 5.0, S8 Fig).

**Table 2:** Clinical and demographic characteristics of AoU T2DM cases (n = 16,915) compared with controls (n = 16,915). Values are shown as mean ± standard deviation or percentage. P-values were determined by a two-sample t-test. “NA” indicates data were not applicable or unavailable for controls. Maximum values were used for Laboratory Measurements if multiple values were available.

Characteristic	T2DM Cases (n = 16,915)	Controls (n = 16,915)	P-Value
**Demographics**			
Age (y), Mean ± SD	69.5 ± 11.1	75.9 ± 6.4	<0.0001
Male, %	50.9%	50.9%	1.0
Age at Diagnosis (y), Mean ± SD	61.9 ± 11.0	NA	NA
**Anthropometry**			
BMI (kg/m^2^), Mean ± SD	33.8 ± 7.7	31.0 ± 5.8	<0.0001
**Laboratory Measurements**			
HbA1c (%), Mean ± SD	7.7 ± 2.2	5.1 ± 1.1	<0.0001
Glucose (mg/dL), Mean ± SD	226.0 ± 105.1	126.2 ± 40.2	<0.0001
Total Cholesterol (mg/dL), Mean ± SD	202.1 ± 64.6	205.1 ± 55.6	0.002
Low-Density Lipoprotein (mmol/L), Mean ± SD	3.1 ± 0.9	3.7 ± 0.9	<0.0001
High-Density Lipoprotein (mg/dL), Mean ± SD	117.4 ± 49.6	121.6 ± 44.4	<0.0001

**Fig 4:**
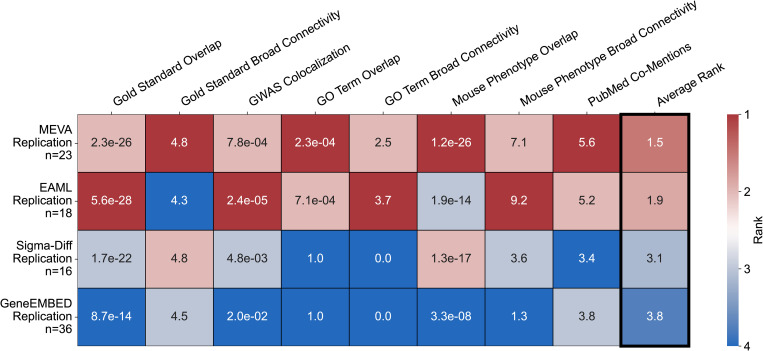
Genes replicated by MEVA across UKB and AoU are most related to diabetes biology. Heatmap displaying replicated gene performance across multiple validation criteria. MAGMA was omitted as no genes were replicated. Cells are color-coded by rank (red = best, blue = worst) and annotated with either p-values (GWAS Colocalization, Gold Standard, GO Term, and Mouse Phenotype Overlap) or z-scores (Gold Standard Broad Connectivity, GO Term and Mouse Phenotype Broad Connectivity, and 1 + PubMed Co-Mentions). No method reached significance for Gold Standard Direct Connectivity and was omitted.

Next, we assessed cross-cohort replication. Among the 99 AoU genes, 23 overlapped with the 101 UKB genes (p = 1.6e-32; Table 3), including 11 gold standards. To contextualize this finding, we benchmarked this performance against the other methods. In this comparison, GeneEMBED replicated the most genes (n = 36; p = 1.4e-34), followed by MEVA, EAML (n = 18; p = 1.2e-17), and Sigma-Diff (n = 16; p = 2.3e-15), while MAGMA failed to replicate any genes, although its input variant definitions changed across cohorts. To determine if a higher replication count equated to greater biological relevance, we repeated our validation analyses and found that MEVA’s 23 genes were the most related to T2DM biology, outperforming all other sets, including the larger set from GeneEMBED (Fig 4). These 23 high-quality replicated genes, including gold standards (*WFS1, SLC30A8, HNF1A*) and less-characterized candidates (*ADAM30, NRIP1, CALCOCO2, TUBB1, WDR90, ZFP36L2*), are further supported by broadening the AoU threshold to nominal significance (p < 0.05), which increased the overlap to 48 genes (S12 Table), capturing four additional gold standards (*COBLL1, GCK, PPIP5K2, GIPR*). Taken together, these data demonstrate that MEVA identifies a robust set of candidates with the highest biological relevance, and that this signal of cross-cohort consistency extends to a wider group of nominally significant genes.

**Table 3:** MEVA genes replicated in UKB and AoU (n = 23). Genes are annotated with the MEVA p-value and rank (1 = most significant) in UKB and AoU, and sorted by the number of PubMed co-mentions between the gene and “Type 2 Diabetes”.

Gene	UK Biobank		All of Us		PubMed Co-Mentions with “Type 2 Diabetes”
	Rank	P-Value	Rank	P-Value	
*HNF1A*	3	5.60E-17	6	5.00E-16	637
*PPARG*	17	1.60E-13	23	1.90E-10	317
*KCNJ11*	43	5.00E-09	24	2.90E-10	314
*SLC30A8*	1	<5.6e-17	26	3.30E-10	308
*ABCC8*	34	4.60E-10	52	5.70E-08	237
*WFS1*	1	<5.6e-17	9	4.30E-15	107
*MLXIPL*	73	2.10E-06	76	1.70E-06	71
*NEUROG3*	39	2.30E-09	70	9.60E-07	43
*PAM*	3	5.60E-17	95	6.30E-06	39
*THADA*	3	5.60E-17	39	7.40E-09	38
*LGALS3*	3	5.60E-17	1	5.60E-17	28
*ARAP1*	3	5.60E-17	27	4.20E-10	20
*COMT*	19	4.90E-13	31	1.70E-09	19
*EPHX2*	28	1.50E-10	21	1.50E-10	15
*MCL1*	97	7.50E-06	86	3.10E-06	12
*NRIP1*	3	5.60E-17	25	3.20E-10	8
*NCAN*	32	3.20E-10	17	1.40E-11	8
*GPSM1*	44	7.60E-09	12	1.20E-13	3
*ADAM30*	36	7.90E-10	60	3.20E-07	2
*CALCOCO2*	33	4.60E-10	79	2.00E-06	1
*ZFP36L2*	101	9.20E-06	64	5.60E-07	1
*TUBB1*	53	4.60E-08	72	1.10E-06	0
*WDR90*	57	1.30E-07	1	5.60E-17	0

To assess whether these shared genes exhibited consistent risk modification, we calculated gene-level odds ratios (OR) stratified by EA scores, leveraging prior work linking EA 30–70 to gain-of-function (GOF) and EA 70–100 to loss-of-function (LOF) [26] (S13 Table). Using all variants (AF < 50%), several genes showed same-direction risk effects across both cohorts, including the gold standards *PAM* (EA 30–70; UKB OR = 1.13, 95% Confidence Interval (CI) = 1.07-1.32e-6, FDR = 1.5e-5; AoU OR = 1.11, 95% CI = 1.04 – 1.18, p = 0.001) and *ARAP1* (EA 30–70; UKB OR = 0.91, 95% CI = 0.88-0.94; FDR = 1.0e-7; AoU OR = 0.93, 95% CI = 0.89 – 0.969, p = 7.3e-4), and three less-characterized genes: *NRIP1, TUBB1,* and *CALCOCO2*. First, potential LOF variants in *NRIP1* increased risk in both UKB (OR = 1.09, 95% = CI 1.04–1.13, FDR = 5.4e-4) and AoU (OR = 1.09, 95% = CI 1.03–1.15, p = 0.002), largely driven by p.Arg448Gly. Removing this variant and restricting the analysis to rarer alleles (AF < 5%) strengthened the UKB signal (OR = 1.27, 95% CI = 1.02-1.60, p = 0.039), but rendered the AoU association non-significant (OR = 0.97, 95% CI = 0.74 – 1.27, p = 0.89), suggesting a potential LOF model for *NRIP1* in T2DM*.* Second, potential GOF variants in *TUBB1* associated with increased risk in UKB (OR = 1.07, 95% CI = 1.01–1.13, FDR = 0.04) and AoU (OR = 1.09, 95% CI = 1.02–1.17, p = 0.01). After excluding the common allele p.Gln43Pro, the rare-variant signal strengthened in AoU (OR = 1.56, 95% CI = 1.06-2.29, p = 0.03) and remained significant in UKB (OR = 1.07, 95% CI = 1.01-1.13, p = 0.02), suggesting a GOF-T2DM risk model for *TUBB1*. Third, potential GOF variants in *CALCOCO2* associated with increased risk in both UKB (OR = 1.05, 95% CI = 1.02–1.09, FDR = 0.02) and AoU (OR = 1.05, 95% CI = 1.01–1.10, p = 0.02), driven by p.Gly164Glu. In contrast, rare variants showed non-significant protective effects (UKB: OR = 0.93, 95% CI = 0.79-1.09, p = 0.39; AoU: OR = 0.92, 95% CI = 0.74 – 1.13, p = 0.45), consistent with a LOF model previously linked to β-cell insulin content [42]. Lollipop plots showing each variant and its position along the protein sequence are provided in S9 Fig. This trend of consistent directionality extended to the 25 nominally replicated genes. For potential GOF variants, 17 of 25 (68%) showed the same effect direction, and for potential LOF variants, 15 of 25 (60%) had the same effect direction. In total, five of these genes reached statistical significance in both cohorts, reinforcing signals for established genes like *GCK* and *GIPR,* and, more importantly, identifying three completely novel candidates with cross-cohort protective effects: *H1FNT* (UKB OR=0.92 (0.89-0.95), FDR = 3.4e-7; AoU OR=0.95 (0.91-1.0), p = 0.04), *ASCC2* (UKB OR=0.90 (0.86-0.94), FDR = 7.3e-7; AoU OR=0.92 (0.87-0.98), p = 0.007), and *ZNF641* (UKB OR=0.91 (0.88-0.94), FDR = 7.4e-8; AoU OR=0.95 (0.91-0.99, p = 0.01). These consistent risk effects across cohorts support shared pathophysiological mechanisms, reinforcing these genes as credible contributors to T2DM risk.

**Fig 5:**
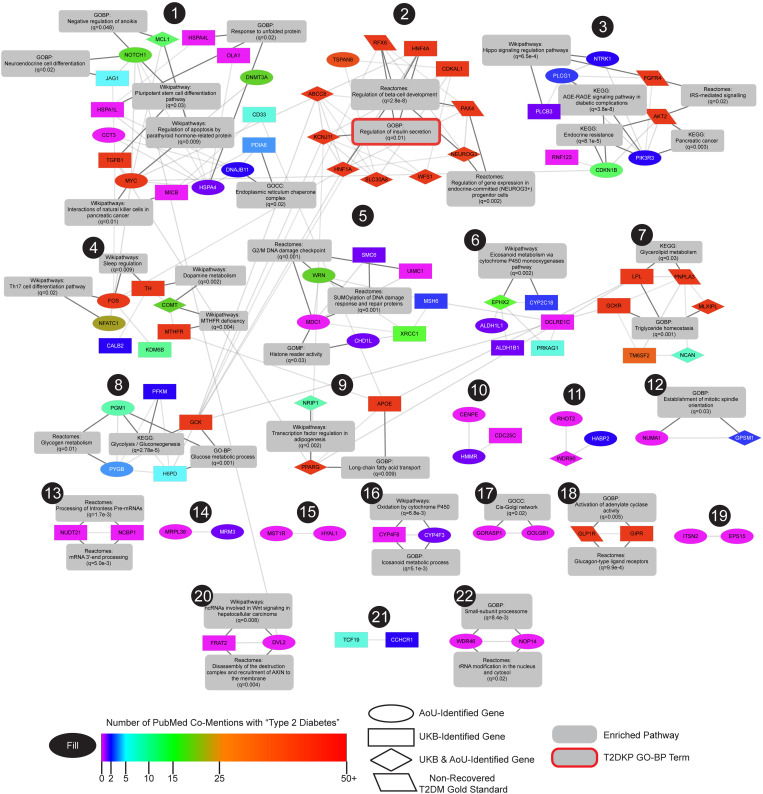
UKB and AoU MEVA genes cluster in the STRING network. MEVA candidates and T2DM gold standards (parallelograms) clustered in the STRING v12 network (edge weight > 0.7). MEVA node shapes represent the cohort in which they were discovered (ovals = AoU-specific, rectangles = UKB-specific, diamond = cross-cohort genes). If MEVA identified a gold standard, it is drawn in the shape of the cohort in which it was rediscovered. Nodes are filled by the PubMed co-mentions with “Type 2 Diabetes” (few in magenta to many in red). Clusters are labeled with selected enriched pathways (q < 0.01) and outlined in red if included in the T2DKP GO-BP set.

Beyond gene-level replication, we asked whether the AoU and UKB MEVA candidates, when combined, were enriched in shared diabetes-related pathways. We clustered the 31 gold standards, 101 UKB- and 99 AoU-candidates in STRING and performed cluster-specific enrichment analysis (Fig 5 and S14 Table). Several clusters revealed cross-cohort pathway convergence. In cluster one, the largest cluster without gold standards, we observed enrichment for *endoplasmic reticulum (ER) chaperone complex* (q = 0.02; *PDIA6* [UKB], *DNAJB11* [AoU]), which mitigates ER stress in β-cells [53], and *neuroendocrine cell differentiation* (q = 0.02; *NOTCH1* [AoU], *JAG1* [UKB]), known to modulate β-cell activity [54]. Cluster three was enriched for *Hippo signaling regulation pathways* (q = 8.5e-4; *FGFR4* [gold standard], *PLCB3* [UKB], *NTRK1* [AoU]), implicated in β-cell growth and apoptosis [55]. Cluster eight highlighted *glycolysis/gluconeogenesis* (*q* = 2.8e-5; *PFKM* [UKB], *PGM1* [AoU]) and *glucose metabolic process* (*q* = 0.001; *H6PD* [UKB], *GCK* [UKB], *PGM1* [AoU]), both central to glucose homeostasis. Together, the results show that MEVA not only replicates gene-level signals, but also converges on shared diabetes pathways, spanning established and newly implicated genes, thereby expanding the pool of plausible contributors to T2DM risk.

### Multidimensional prioritization reveals new T2DM risk genes

**Fig 6:**
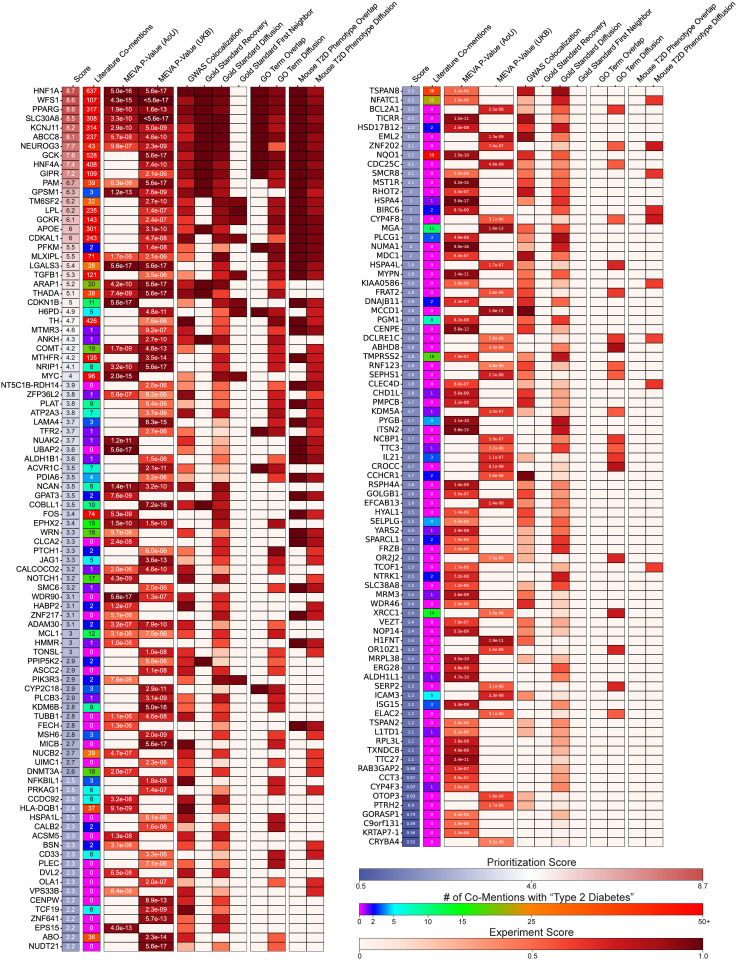
Functional prioritization of MEVA candidates. Heatmap ranking 177 MEVA candidate genes by integrated validation evidence. Prioritization scores from highest (8.7; red) to lowest (0.5; blue). PubMed co-mentions with “Type 2 Diabetes” range from 0 (magenta) to 50+ (red). Evidence categories (robustness, genetic, pathway, phenotype) are scored from 0 (no evidence; white) to 1 (strong evidence; dark red).

To guide experimental follow-up, we prioritized all 177 MEVA candidates from the UKB and AoU cohorts using a ten-metric scoring system integrating four functional domains: statistical robustness, genetic support, pathway association, and phenotypic relevance. Scores ranged from 0.52 (*CRYBA4*) to 8.7 (*HNF1A*; Fig 6 and S15 Table) and provide a structured, biologically informed framework to prioritize candidates. The prioritization revealed four tiers. First, gold standard T2DM genes (e.g., *HNF1A, WFS1, PPARG*) received top scores, thus validating our approach. Second, several UKB-specific candidates with limited literature evidence scored near the top of the list, which may reflect UKB’s longer availability and greater opportunity for follow-up studies to accumulate supporting evidence. Examples include *PFKM* (score = 5.5; 2 co-mentions), *H6PD* (4.9; 5 co-mentions), and *MTMR3* (4.6; 1 co-mention). Among these, *PFKM* was the top non-gold-standard gene with limited literature support. Its score was driven by enrichment in T2DM-relevant pathways, such as *regulation of hormone secretion,* and phenotypes, including *abnormal glucose homeostasis*. The other UKB-specific candidates showed similar functional support, positioning them as credible, previously overlooked contributors to disease biology. Third, AoU-specific genes generally scored lower, which may be attributed to AoU’s more recent establishment and limited opportunities for follow-up studies. Nonetheless, genes with limited literature evidence, such as *NUAK2* (3.7; 1 co-mention) and *UBAP2* (3.6; 0 co-mentions), emerged as promising leads. Notably, *UBAP2* ranked fourth in AoU and *NUAK2* ranked 15^th^, with both linked to the *increased circulating glucose level* mouse phenotype. Fourth, among the 23 replicated genes, a subset including *NRIP1* (4.1; 8 co-mentions), *ZFP36L2* (3.8; 1 co-mention), *CALCOCO2* (3.2; 1 co-mention), *ADAM30* (3.1; 2 co-mentions), *WDR90* (3.1; 0 co-mentions), and *TUBB1* (2.8; 0 co-mentions), emerged as under-characterized yet consistently supported candidates. For example, *ZFP36L2* colocalized with many *THADA*-mapped SNPs and was linked to the T2DM-related mouse phenotype *decreased splenocyte number*. While these genes ranked mid-range overall, they replicated across populations and exhibited modest functional associations, positioning them as high-confidence, novel targets for discovery. This prioritization framework systematically stratifies MEVA’s candidates based on independent functional validations, providing a practical roadmap for experimental validation and a new blueprint for understanding the genetic architecture of T2DM.

## Discussion

Despite extensive progress, the catalog of T2DM genetic risk factors remains incomplete. One reason is that conventional methods limit the scope of allele frequencies and fail to incorporate the predicted functional impacts of variants. We addressed these gaps with MEVA, a framework that unifies three complementary, gene-level association tests that account for the functional impact of coding variants. Applied to 28,115 T2DM UKB exomes, MEVA identified 101 risk genes, 95 of which were not prioritized by MAGMA. Further, MEVA outperformed its component methods and MAGMA across genetic, pathway, phenotypic, and robustness validation criteria, even after removing well-established genes. In an independent analysis of 16,915 AoU exomes, MEVA identified 99 genes. These included 23 genes replicated across cohorts, while the remaining cohort-specific genes, when combined, were also enriched in shared diabetes-related pathways, thereby nominating new candidates to expand T2DM genetic architecture beyond well-established loci.

Weighting protein-coding variants by their predicted functional impact, rather than treating all alleles as equal, enhances the detection of biologically relevant gene-disease associations. Our previous work in cancer [26,27], early-onset coronary artery disease [28], autism [29], and Alzheimer’s [30–32] demonstrates this, and, here, we extend that body of evidence to T2DM. All three component EA methods outperformed the impact-agnostic MAGMA across genetic, pathway, phenotypic, and robustness validation criteria – even when MAGMA’s input included non-coding variants. These findings suggest that the substantial information gained from integrating the functional impact of coding variants can outperform the information lost by excluding non-coding variants. This may reflect the inherent difficulty of correctly assigning non-coding effects to their target genes. While newer methods exist that may better integrate these non-coding effects [56], MAGMA remains a widely used gene-based test and serves as a robust benchmark. These results support a broadly applicable strategy: integrating variant impact estimates, whether via EA or another predictor, can enhance the precision and biological relevance of gene discovery in complex traits.

Different association tests often detect complementary signals. For example, recent rare-variant exome approaches have prioritized *MAP3K15* [15,16], while previous GWAS have overlooked this association. Thus, combining such methods can improve performance by weighing evidence across tests, amplifying consistent or exceedingly strong signals while suppressing discordant ones. In MEVA, we unified three methods using the CCT, which retains power even when the inputs are correlated. While CCT has been used previously in tools like CauchyGM [35], its application within MEVA yielded notable gains: it ranked gold standard genes higher than individual methods, improved the discovery of biologically supported candidates while filtering out potential false positives with weaker functional support, and maintained accuracy even in smaller cohorts. We note, however, that some gold standards, such as *TPCN2, AKT2,* and *GLP1R*, received lower rankings in MEVA, particularly in larger cohorts. As sample size increases, coding variant impact differences between cases and controls may diminish, resulting in weaker signal and lower gene rankings. This could indicate either no true association or one driven primarily by non-coding variation. Given mixed coding and non-coding GWAS signals near these genes [18,57,58], we hypothesize the latter – that these genes’ associations are driven by non-coding regulatory mechanisms. While MEVA cannot currently test non-coding effects, this pattern may reflect biological complexity that becomes more apparent at scale.

Our analysis identified 23 replicated genes, but a direct comparison of replication performance across methods revealed that the biological quality of these genes is more critical than the raw count. Although the network-based method GeneEMBED replicated more genes, our validation analyses showed that the 23 genes replicated by MEVA were the most enriched for T2DM-related biology. This signal was further supported by a nominal significance threshold, which doubled the number of overlapping genes to 48, and by the enrichment of non-replicated genes in shared, diabetes-relevant pathways, suggesting MEVA is uncovering a mechanistically relevant network of risk factors. Among these 23 replicated genes, three less-characterized candidates, *NRIP1, TUBB1*, and *CALCOCO2*, exhibited same-direction risk effects across both cohorts and were supported by plausible mechanistic models, even in cases where their associations diverged from prior studies. These instances suggest potential context-dependent effects, which are increasingly recognized in T2DM pathophysiology [59], which MEVA may detect through its use of functional impact predictions. For *NRIP1*, previous knockout model studies report improved metabolic health [60,61], but the high-EA, potential LOF variants we observe were associated with increased risk, both at the common (OR = 1.09) and rare variant level (OR = 1.27). This discrepancy could reflect either partial LOF effects (e.g., haploinsufficiency or dominant-negative activity) that fail to activate compensatory pathways seen in the complete knockout, or that a subset of the predicted LOF variants may be hypermorphic, thereby elevating *NRIP1* activity and worsening metabolic control. Both scenarios call for targeted assays to resolve *NRIP1*’s role in diabetes. Potential GOF variants in *TUBB1,* which encodes the platelet β-tubulin isoform and has no prior connection to T2DM, showed risk-increasing effects (OR ≈ 1.08). *TUBB1* has been linked to worsened liver fibrosis outcomes [62], implicating it in hepatic physiology and, by extension, glucose homeostasis. Similarly, GOF variants in *CALCOCO2,* a coiled-coiled protein involved in autophagy, are also associated with increased risk (OR = 1.05), with rare variants trending protective (OR ≈ 0.92), a pattern that mirrors β-cell insulin-granule loss observed in knockout models [42].

MEVA identified three additional cross-cohort candidates, *ADAM30, ZFP36L2,* and *WDR90*, with limited literature evidence and plausible biological mechanisms. First, *ADAM30* colocalizes with ten T2DM-associated SNPs previously attributed to *NOTCH2* [18,63,64] and participates in the *primary metabolic process* GO-BP term. Despite its initial characterization as testes-specific, its expression in adipose tissue [65] and association with fasting glucose [66] support a more central metabolic role. Second, *ZFP36L2*, a tristetraprolin-family protein, colocalizes with many SNPs attributed to the gold standard *THADA* [18,63,67,68] and is linked to the mouse phenotype *decreased splenocyte number*. Prior research has implicated the spleen in regulating insulin secretion [69,70], suggesting a role within the immune-metabolic axis. Finally, *WDR90,* a gene with no prior literature on T2DM and minimal functional characterization elsewhere, was the most significant gene in the AoU cohort and colocalized with SNPs assigned to neighboring genes (*FAM234A*, *LMF1)* [18,63]. *WDR90* is implicated in ciliogenesis [71,72], and recent studies link β-cell primary cilia to insulin secretion dynamics [73], raising the possibility that *WDR90* may influence cilium-dependent insulin secretion. These replicated, yet under-characterized genes represent credible new contributors to T2DM genetic architecture.

In addition to replicated genes, MEVA identified cohort-specific candidates with strong biological relevance. From the UKB-specific candidates, *PFKM***,** encoding the muscle isoform of phosphofructokinase, is often linked to insulin resistance and secretion [74,75], but has limited literature tying it to T2DM directly. In our prioritization, *PFKM* was the top-ranked non-gold standard (score = 5.5), supported by pathway enrichment for *regulation of hormone secretion* and many mouse phenotypes, like *impaired glucose tolerance*. *H6PD*, encoding hexose-6-phosphate dehydrogenase, is differentially expressed in T2DM patients [76], may protect against obesity [77], and supports redox balance [78], potentially mitigating oxidative stress-driven metabolic disturbance. Together, *PFKM* and *H6PD* drove enrichment for the mouse phenotype *increased skeletal muscle glycogen level* (*q* = 3.6e-4), suggesting a shared role in peripheral glucose homeostasis. From the AoU candidates, *UBAP2,* a ubiquitination-pathway component, ranked 4^th^ by p-value and is noted to reduce pancreatitis in knockout mice [79], suggesting a potential link to β-cell stress through pancreatic inflammation, a known contributor to diabetes risk [80]. *NUAK2* is involved in glucose-starvation sensing and Hippo signaling and reduces hyperglycemia when up-regulated [81], suggesting a therapeutic role in T2DM. Both *NUAK2* and *UBAP2* linked to the *increased circulating glucose level* mouse phenotype, providing *in vivo* evidence of their metabolic involvement. While these genes were identified in only one cohort, their functional relevance, phenotypic enrichment, and therapeutic potential support them as credible contributors to diabetes risk. As the AoU cohort continues to grow and is further explored, we expect supporting evidence for AoU-prioritized genes to accumulate and reflect their potential more fully.

While the replication of 23 genes was highly significant, the incomplete overlap warrants examination. The observed replication rate is likely influenced by key differences between the cohorts. First, our QC strategies differed between cohorts. We followed established practices for the well-characterized UKB data [16] but applied more stringent thresholds to the newer AoU data to prioritize specificity over sensitivity. As AoU becomes more utilized, we expect the field to coalesce around optimal thresholds. Further, the use of different sequencing technologies, single-site whole-exome sequencing (WES) in the UKB versus multi-site short-read whole-genome sequencing (srWGS) in AoU, may affect variant quality and comparability. Biologically, the cohorts likely represent distinct local ancestries. The UKB cohort exhibits a more homogenous British-Irish ancestry, while AoU likely represents diverse European ancestries [82,83]. Finally, our inclusion of rare variants, which are more likely to be population-specific, differs from GWAS that benefit from more replicable common variants, especially when using standardized genotype arrays like MetaboChip [84,85]. In addition to these cohort-level factors, the modest replication is also an expected consequence of reduced statistical power in the smaller AoU cohort, which in turn revealed a key methodological trade-off between replication consistency and biological specificity. GeneEMBED’s network-based approach was the most resilient, replicating 36 genes, but this set was less specific to T2DM biology. In contrast, the purely statistical method EAML replicated fewer (n = 18), but more T2DM-specific genes, highlighting MEVA’s strength in balancing these factors to produce a replicated set that is the most significantly related to diabetes. Therefore, while our replication rate was highly significant, these cohort-specific differences highlight that future harmonization of QC and ancestry-matching may improve cross-study replication.

Our study’s interpretation rests on several key assumptions, each with resulting limitations that we sought to mitigate. First, we assumed our cohort and phenotype definitions were robust to identifying T2DM-associated signals. By restricting our analysis to individuals of European ancestry and treating T2DM as a single condition, the generalizability of our findings to other ancestries [86] and clinical subtypes [63,87–89] may be limited. However, our down-sampling experiments demonstrated that MEVA remains accurate in substantially smaller cohorts, providing strong support for its use in other ancestries and sub-populations. Further, our reliance on electronic health records and self-reported data for case/control definitions carries a risk of patient misclassification. We attempted to mitigate these challenges with stringent filtering, the selection of older controls to ensure more probable non-diabetic status, and ultimately by demonstrating the replication of 23 genes in the independent AoU cohort. Second, our framework assumes the accurate measurement of coding variant effects. MEVA’s current scope is limited to protein-coding variants, omitting non-coding regions known to influence T2DM [90–95]. It also assumes that EA scores accurately quantify functional impact and that our gain/loss-of-function binning is appropriate. The validity of this EA-based approach is strongly supported by each method’s superior performance over MAGMA in this study and its successful application to other complex diseases [26–32]. Finally, our validation process assumed accuracy across our external benchmarks. Our gold standard gene set and the external databases used (e.g., STRING, MGI) are notably biased towards well-studied biology, a limitation which could down-weight truly novel genes in our prioritization. We anticipated this by designing a multi-metric prioritization framework that buffers against this bias by considering evidence from multiple, independent domains.

In summary, MEVA provides a scalable and generalizable framework that enhances gene discovery by integrating the functional impact of protein-coding variants. In applying it to two large, independent biobanks, we outperformed conventional methods, identified and replicated novel T2DM candidate genes, and revealed convergence on key biological pathways. This work not only nominates candidates to expand the catalog of genetic risk factors for T2DM but also provides a functional, prioritized roadmap to guide future experimental and therapeutic research.

## Materials and methods

### UK Biobank T2DM cohort and exome quality controls (QC)

To assemble case and control samples, we used 469,789 whole exomes available in the UKB (application no. 55532), a prospective study reporting health records, self-reported surveys, blood biomarkers, and other clinical data. Based on phenotypic data downloaded April 2024, we aggregated two patient groups: 1) T2DM cases (n = 28,542), defined by E11 ICD-10 codes diagnosed after age 35, with exclusions for type 1 diabetes mellitus (T1DM) and missing HbA1c or BMI values; 2) unaffected, healthy controls (n = 191,019), excluding samples with T1DM or T2DM, with further exclusions for family history of diabetes or pre-diabetic HbA1c levels (>5.6%) at intake, and self-reported serious medical conditions, to broadly minimize confounding from comorbidities (S1 Table). We performed QC and removed potential false-positive variants using widely established thresholds [16], which were refined by visual inspection of the data distributions. The applied filters included read depth (DP) below 10, genotype quality (GQ) below 20, missingness exceeding 5%, and Hardy-Weinberg equilibrium exact test p-value below 5e-8. We mapped the remaining variants to GRCh38 canonical transcripts in MANE [96] using Ensembl’s Variant Effect Predictor v94 and annotated EA scores. Next, we applied sample filters: 1) ancestry principle component (PC) analysis to exclude non-European White ancestries (Probability < 0.85); 2) X chromosome heterozygote/homozygote ratios to exclude sex mismatches (Male > 0.0005, Female < 0.0005); 3) Somalier [97] relatedness (v0.2.16) to exclude samples with kinship coefficients above 0.2; and 4) removal of sequencing statistic outliers, including total variants, transition/transversion ratio, singletons, missing calls, heterozygous/homozygous ratios, and indels. These steps yielded 28,115 T2DM cases and 181,967 potential controls. Finally, we defined our cohort of T2DM cases (n = 28,115) and randomly selected an equal number of healthy controls from the eligible pool (n = 181,967), filtering controls to be older than 69 (70% of T2DM cases were diagnosed by 69). The two groups were matched 1:1 by sex and by distributions of ancestry PCs 1–5 (Kolmogorov-Smirnov (KS) p > 0.05).

### All of Us T2DM validation cohort

To aggregate a validation cohort, we assembled European White, non-Hispanic samples from 414,830 All of Us (AoU) version 8 participants, accessed in February/March 2025. AoU is a prospective study of diverse U.S. adults integrating genomic, clinical, and lifestyle data. We identified 18,176 T2DM cases (S10 Table) via our previous T2DM case definition, modified to include participants with either HbA1c measurement or an anti-diabetic prescription (S11 Table) to account for missing values. We selected 41,156 healthy controls aged ≥ 66 years, using similar UKB cohort phenotypic filters. These filters were adapted from the UKB criteria to account for differences in data availability and population distributions. We then extracted exome-restricted variant calls from the All of Us srWGS Hail matrix (Gencode v42 basic transcripts ± 15 bp) and applied QC filters to exclude low-quality variants. We based our AoU quality control on the AoU Researcher Workbench guidelines [98], modified to be more stringent to prioritize specificity. This approach was chosen to ensure a high-confidence variant set from this newer, more heterogeneous cohort. The applied filters include: GQ < 20, call rate < 0.99, allele number < 0.95, HWE ≤1e-8 and potentially problematic samples, using pre-generated auxiliary files. We removed samples with low quality (flagged_samples.tsv), < 80% European ancestry probability (ancestry_preds.tsv), high relatedness (relatedness_flagged_samples.tsv), and sequencing outlier metrics computed from the filtered matrix table (total variants, singleton count, heterozygous/homozygous ratio, transition/transversion ratio, indels). Finally, we defined our T2DM case cohort (n = 16,915) and then selected an equal number of healthy controls at random from the eligible group (n = 41,156), stratifying by sex and ancestry PCs, following the same UKB stratification procedure (Table 2). For the non-coding-inclusive MAGMA analysis, we retrieved the full AoU WGS variant set from the Hail Variant Dataset, applied the same sample- and variant-level QC as above, and used MAGMA’s gene-annotation step to map coding and intronic variants plus ±10 kb upstream/downstream flanks to genes [38].

### Sigma-diff analysis

For each gene *i*, we quantified its aggregate mutational burden in a cohort by summing the EA values of all protein coding variants carried by every case and control, then normalized by gene length to obtain a per-residue “mutational energy” *E*_*i*_*.* This raw burden is rescaled to a dimension-less score, $\sigma_{i},$ derived from the law of equipartition, which states that at equilibrium, the mutational energy per residue should be uniform when normalized by gene length [23,99,100]. Each gene is considered an independent evolutionary unit under weak coupling, yielding:


$$\sigma_{i}=\;\frac{L_{i}\;E}{L\;E_{i}}$$ (1)

where *L*_*i*_ is the length of gene *i*, *E* is the total energy summed across the genome for all individuals, and *L* is the total of proteomic length, calculated as the sum of all gene lengths ($\sum_{i}L_{i}$) using the largest transcript per gene in the MANE/Ensembl annotation. Sigma-Diff computes the difference between controls and cases as:


$${\Delta \sigma }_{i}=\sigma_{i}^{controls}-\;\sigma_{i}^{cases}$$ (2)

The significance of ${\Delta \sigma }_{i}\;$is assessed by permuting case-control labels 1,000 times to generate an empirical null distribution and a two-sided *z*-score. Positive Z-scores indicate higher mutational burden in cases, suggesting a potential causative role, whereas negative Z-scores suggest higher burden in controls, implying a potential protective effect. Genes with |*z*| > 3 are deemed significant, yielding the Sigma-Diff candidate set.

### MEVA pipeline analysis

To identify genes with abnormal functional impact distributions in cases and controls, we analyzed each alternate allele at protein-coding sites, including only those with allele frequency ≤ 50% and consequence types of missense, stop-gained, frameshift, stop-lost, splice-donor, and splice-acceptor. For multiallelic sites, only alternate alleles meeting both the frequency and consequence criteria were retained. We assigned a maximal score (100) to estimated loss-of-function variants (stop-gained, frameshift, stop-lost, splice-donor, splice-acceptor) and scaled missense variants from 0-100 based on EA. We analyzed these variants using the three MEVA component algorithms: EAML, Sigma-Diff, GeneEMBED. EAML uses an ensemble of nine machine learning classifiers, learning over 10x cross-validation and six EA-thresholded dominant/recessive features to identify genes that maximally separate cases and controls. GeneEMBED adjusts a predefined protein interaction network (STRING v12) by weighting edges with the sum of EA scores from each node, generating averaged networks for cases and controls, where the node embedding distance between these networks reflects each gene’s functional impact. Because GeneEMBED can up-rank network hub nodes [32], we performed a control versus control analysis to identify and exclude any overlapping genes from the T2DM candidate list. No overlaps occurred for EAML or Sigma-Diff. Genes meeting a FDR ≤ 0.1 (EAML, GeneEMBED) or |*z|* ≥ 3 (Sigma-Diff) threshold were considered significant.

To integrate our algorithms, we selected CCT over consensus or multiplicative approaches due to its accommodation of correlated p-values [33]. CCT computes a weighted sum of transformed p-values using the formula:


$$T=\;\sum_{i=\text{1}}^{d}\omega_{i}\text{tan}\{\left(\text{0}.\text{5}-p_{i}\right)\pi \}$$ (3)

Given that there are d methods, let $p_{i}$ represent the p-value for the method i where i=1, 2, …, d  and the weights $\omega_{i}\geq \text{0},\;\forall i$ and $\sum_{i=\text{1}}^{d}\omega_{i}=\text{1}$. The transformation $\text{tan}\{\left(\text{0}.\text{5}-p_{i}\right)\pi \}$ follows a standard Cauchy distribution, assuming that the $p_{i}\sim \text{Uniform}(\text{0},\;\text{1})$. Consequently, this property ensures that the tail of *T*’s null distribution remains approximately Cauchy even under arbitrary correlations, a critical advantage given the application to the same variant set. Thus, the combined p-value is calculated by:


$$p_{cauchy}=\text{0}.\text{5}-\;\frac{arctan(T)}{\pi }$$ (4)

Because the arctan function (Numpy v1.24.4) uses the standard 64-bit floating-point format, any p-value smaller than 5.6e-17 falls below the precision limit and is rounded to zero. We therefore report such values as “<5.6e-17”, indicating that the true p-value lies below this numerical limit, but remains greater than zero. To address overlapping GeneEMBED signals, we masked p-values for control-control identified genes and combined p-values from EAML and Sigma-Diff. We applied a gene-level significance threshold of p < 1e-5 for three reasons: 1) a strict Bonferroni correction (p < 2.8e-6) eliminated roughly 20% of the biologically coherent hits, limiting discovery [101]; 2) with ~18,000 genes, p < 1e-5 corresponds to roughly 0.2 expected false-positives per scan, a rate that was further controlled by independent replication; and 3) the p < 1e-5 threshold has been successfully applied in other exome-wide studies of complex traits [102,103].

### MAGMA analysis

We benchmarked MEVA against a conventional impact-agnostic test with MAGMA. We analyzed variants with MAF ≤ 0.5, adjusting for sex as a covariate. We adopted a suggestive significance threshold of p ≤ 0.01. This threshold is consistent with suggestive thresholds used in similar gene-based tests like SKATO [40,41] and generates a candidate list of a comparable size to the other methods, allowing for a more fair performance comparison.

### Criteria for success

#### Gold standard construction.

To compile a set of high-confidence T2DM “gold standards”, we extracted T2DM-associated genes (MONDO_0005148) from the Open Targets Platform “target-disease association” resource, which integrates diverse evidence streams, but, notably omits the STRING network [104]; thus, benchmarking GeneEMBED remains non-circular. We identified 7,700 genes and filtered for an “Overall Association” and a “Genetic Association” above 0.4, yielding 159 genes. Next, we selected coding-variant-driven genes with missense SNPs in GWAS or rare-variant burden signals, resulting in 31 genes (S2 Table). Direct overlap was assessed using a one-tailed hypergeometric test.

#### Gold standard ranking.

We hypothesized that MEVA would rank T2DM gold standards higher than its component methods and MAGMA. To test this, we ranked all genes by their raw p-values and calculated the area under the receiver operator curve (AUROC) for recovering the 31 gold standards.

#### GWAS loci co-localization.

We hypothesized that the candidate genes share genomic proximity with known T2DM GWAS loci, indicating potential causality. We retrieved T2DM (MONDO_0005148) GWAS loci from the GWAS Catalog API (p < 5e-8) in January 2025 and mapped genes using GRCh38 v94 coordinates. Genes were considered co-localized if any part fell within 0.5 Mbp of a GWAS locus. Significance was determined by Fisher’s exact test against background genes from GRCh38 v94.

#### Network connectivity.

We hypothesized that our candidate genes share protein interactions with known T2DM risk genes, suggesting shared biological functions. First, we tested first neighbor connectivity by counting edges shared between exclusive candidate genes (excluding recovered gold standards) and 31 T2DM gold standard genes using STRING v12 with highest-confidence edges (weight > 0.9). Significance was determined using a z-test against a null distribution derived from counting edges between 100 degree-matched random gene sets and the gold standards. Next, we tested broad connectivity by applying the network diffusion algorithm [44] to the STRING v12 network (all edges). This algorithm simulates signal diffusion from seed nodes across a graph structure, with signal strength decreasing according to edge weights, ultimately ranking nodes by received signal. We diffused signal from exclusive candidate genes as seeds and measured the ranking of gold-standard genes using AUROC. Significance was determined using a z-test against a null distribution of AUROC scores from 100 degree-matched random gene sets receiving the signal. Z scores > 2 were interpreted to be significant in both of these analyses. These analyses are independent of our initial gene selection since GeneEMBED used only the network structure and did not receive *a priori* T2DM gold standard information.

#### Gene ontology enrichment.

We hypothesized that our candidate genes participated in Gene Ontology Biological Process (GO-BP) terms related to T2DM. First, we used STRING v12’s pathway enrichment tool, via API, considering terms significant at a FDR < 0.05. To compare against T2DM-related pathways, we collected GO-BP terms from the Type 2 Diabetes Knowledge Portal (T2DKP; all ancestries), accessed in February 2025. Direct overlap was assessed with a one-tailed hypergeometric test. Next, we measured GO-BP term similarity by applying the network diffusion algorithm to the GO-BP ontology graph, with each parent-child edge set to a weight of 1. We diffused signal from exclusive candidate-enriched terms as seeds and measured the ranking of T2DKP GO-BP terms using AUROC. We determined significance by a z-test using a null distribution derived from AUROC scores of 100 degree-matched random GO-BP terms sets receiving the signal. Z-scores > 2 were considered significant.

#### Mouse genome informatics (MGI) enrichment.

To test whether our candidate genes link to T2DM-associated *in vivo* phenotypes, we downloaded gene-phenotype associations from MGI via the OTP FTP site in October 2024 and tested enrichment with a z-test by comparing the number of overlapping candidate genes for a phenotype against a null distribution of overlaps from 5,000 random gene sets matched for representation, accounting for the number of mouse models per gene. We transformed the z-scores to p-values and applied an FDR correction, where FDR < 0.05 was considered significant. Next, we extracted 261 T2DM phenotype-disease associations (OMIM: 125853), curated by the Alliance of Genome Resources [49], and measured direct overlap using a one-tailed hypergeometric test. Finally, we tested phenotype similarity by applying the network diffusion algorithm to the mammalian phenotype ontology graph, with parent-child edge weight set to 1. We diffused signal from exclusive candidate phenotypes as seeds and measured the ranking of known T2DM phenotypes using AUROC. We determined significance by a z-test using a null distribution derived from AUROC scores of 100 degree-matched random mammalian phenotype term sets receiving the signal. Z scores > 2 were considered significant.

#### PubMed co-mention enrichment.

We hypothesized that candidate genes are more frequently mentioned in T2DM-related literature (i.e., the occurrence of a gene name and “Type 2 Diabetes” within the same manuscript). To test this, we queried PubMed (January 2025) across all manuscript text using the Bio.Entrez Python package v1.83 [105] with the query: “{gene}” AND “Type 2 Diabetes” AND (“gene” OR “protein”). We calculated enrichment via z-test using null distributions derived from citation counts of 100 random gene sets. We binned genes by citation counts (≥1 and ≥50) and compared the number of candidate genes with *n* citations to the null distribution.

#### Down-sampling.

We hypothesized thatMEVA is more robust than its component methods and MAGMA. To test this, we applied each method to progressively smaller, stratified cohorts, matched by sex and ancestry PCs 1–5, with sample sizes of 500, 1,000, 5,000, 10,000, 15,000, and 20,000 T2DM cases and controls. We generated five random cohorts per sample size and evaluated robustness. First, we computed the hypergeometric overlap between the top 100 genes (ranked by p-value) from each down-sampled experiment and those from the full cohort. Second, we assessed the recovery of 31 T2DM gold standard genes across the full p-value distribution of the down-sampled experiment via AUROC.

#### STRING clustering.

We hypothesized that MEVA would converge on T2DM-related biological pathways between cohorts. To test this, we used STRING v12 with high-confidence interactions (weight > 0.7), embedding 31 T2DM gold standards, 101 UKB- and 99 AoU-identified candidates. Graph clustering was performed using the Markov clustering algorithm (inflation parameter 1.5, optimized for network modularity) contained in the “markov-clustering” v0.0.6.dev0 Python package [https://github.com/guyallard/markov_clustering.git]. We then conducted pathway enrichment analysis for each cluster using Reactomes [106] (downloaded March 2023), GO terms (downloaded Dec. 2024), KEGG [107] (downloaded June 2023), and Wikipathways [108] (downloaded June 2023), assessing overlap using hypergeometric tests and applying an FDR < 0.05 threshold separately across each cluster. This analysis was independent of any a priori T2DM information, including known protein interactions or pathway associations.

#### Odds ratios.

To assess directional effect, we computed sample-wise ORs using a Fisher’s exact test by collapsing variants, thresholded by EA score bins (EA > 30, ≤ 70; EA > 70, ≤ 100) across each of the 23 candidate genes. FDR correction was applied separately across each EA bin.

### Criteria for success-informed ranking of MEVA candidates

To prioritize candidates most likely to hold biological relevance for future functional studies, we ranked genes by aggregating 10 validation experiments grouped into four categories: Robust Signal (MEVA p-value from UKB and Aou), genetic relatedness (gold standard overlap, GWAS co-localization, and STRING first neighbor connectivity and graph diffusion), pathway relatedness (GO-BP overlap and GO graph diffusion) and phenotypic relatedness (mouse phenotype overlap and mammalian phenotype graph diffusion). We scored individual experiments using either binary or inverse rank-based normalization scores (e.g., the gene with the most significant MEVA p-value receives 1, the next 0.99, etc.). Binary scores were assigned 1 if a gene, or its participating pathway/phenotype enrichments, overlapped with our aforementioned set of true positive terms. Inverse rank scoring was used for MEVA p-values, GWAS co-localization, STRING first-neighbor links, and diffusion analyses. Scores were summed for each gene to yield a prioritization score.

### Programming packages

Additional software (not detailed above): pyCFS package v0.1.7.3 (Lichtarge Github), Python v3.8.18, requests v2.31.0, pandas v2.0.3, numpy v1.24.4, matplotlib v3.7.4, matplotlib-venn v1.1.2, Pillow v10.2.0, venn v0.1.3, scipy v1.10.1, network v3.1, biopython v1.83, statsmodels v0.14.1, seaborn v0.13.1, scikit-learn v1.3.2.

## Supporting information

S1 Fig Candidate gene association with 31 T2DM gold standards. Panels A, D, G, J, M, P: Venn diagram showing overlap between each method’s gene list and the gold standards (hypergeometric test). Panels B, E, H, K, N, Q: First-neighbor connectivity of each method’s gene list to gold standards in STRING v12 (edge weight > 0.9). Significance determined by z-test versus 100 random, degree-matched sets. Panels C, F, I, L, O, R: Broad connectivity via network diffusion from each method’s gene list (excluding overlaps) to gold standards in STRING v12 (edge weight > 0). AUROC was calculated by ranking receiving nodes, with significance determined by z-test against AUROC’s of 100 random, degree-matched receiving nodes.
(TIF)

S2 Fig Candidate gene GO-BP enrichment and comparison to T2DKP terms. Panel A: Heatmap of MEVA-prioritized genes versus enriched GO-BP terms (FDR < 0.05), sorted by FDR. Red indicates gene membership in each term. Panels B, D, F, G, I, J: Venn diagrams of overlap between each method’s enriched GO-BP terms (FDR < 0.05) and the 20 T2DKP GO-BP terms (hypergeometric test). Panels C, E, H, K: Broad connectivity via network diffusion from each method’s enriched GO-BP terms (excluding overlaps) to the 20 T2DKP terms with the Gene Ontology structure. AUROC was calculated by ranking receiving terms, with significance determined by z-test against AUROC’s of 100 random, degree-matched receiving GO-BP terms. (Note: Sigma-Diff and MAGMA had no enriched terms and were not included in connectivity analyses).
(TIF)

S3 Fig Candidate gene mouse phenotype enrichment and comparison to T2DM phenotype terms. Panels A, D, G, J, M, P: Enrichment of mouse phenotypes for each method’s gene list. Each point represents a phenotypes -log10(FDR) (y-axis); colored if FDR < 0.05. Phenotypes are grouped into high-level categories (x-axis) and sorted by the number of significant phenotypes. The top five phenotypes are annotated with their label and associated genes. Panels B, E, H, K, N, Q: Venn diagrams between each method’s enriched phenotypes and the 261 T2DM-annotated mammalian phenotypes (hypergeometric test). Panels C, F, I, L, O, R: Broad connectivity via network diffusion from each method’s enriched phenotypes (excluding overlaps) to the 261 T2DM phenotypes in the mammalian phenotype ontology. AUROC was calculated by ranking receiving phenotypes, with significance assessed by z-test against AUROC distributions from 100 random, degree-matched phenotype sets.
(TIF)

S4 Fig Candidate gene co-mentions with “Type 2 Diabetes” in PubMed. For each method’s gene list, we counted how many genes had ≥ 1 co-mention (Panels A, C, E, G, I, K) or ≥50 co-mentions (Panels B, D, F, H, J, L) with “Type 2 Diabetes”. The observed counts are shown as colored dotted lines against a null distribution (gray) generated from 100 random gene sets. Significance was assessed by z-test comparing the observed count to the random distributions.
(TIF)

S5 Fig MEVA-unique genes (n = 40) excel at identifying functionally-related candidates, while EAML-unique genes (n = 98) recover more known associations. Heatmap displaying method performance across multiple validation criteria for genes unique to MEVA and EAML. Cells are color-coded by rank (red = best, blue = worst) and annotated with either p-values (GWAS Colocalization, Mouse Phenotype Overlap) or z-scores (Gold Standard Broad and Direct Connectivity, GO Term and Mouse Phenotype Connectivity, and 1 + PubMed Co-Mentions). Mouse phenotype connectivity and GO term overlap lacked enrichment in either set and were omitted.
(TIF)

S6 Fig MEVA retains superior performance after removal of 31 T2DM gold standard genes. Heatmap displaying method performance across multiple validation criteria for genes significant in MEVA, the component methods, non-prioritized genes, and MAGMA. Cells are color-coded by rank (red = best, blue = worst) and annotated with either p-values (GWAS Colocalization, Mouse Phenotype Overlap) or z-scores (Gold Standard Broad and Direct Connectivity, GO Term and Mouse Phenotype Connectivity, and 1 + PubMed Co-Mentions). Gold standard overlap was removed due to the removal of all gold standards in each method. GO term overlap was removed since no methods overlapped with T2DKP terms. GO term broad connectivity nan values represent no enriched terms to test.
(TIF)

S7 Fig Gold standard rankings across progressively smaller cohorts. Average rankings (1 = most significant) of 31 T2DM gold standards across progressively smaller cohorts. Genes are color coded and sorted in each plot based on the mean rank in the 20k v. 20k experiments (top gene = best ranked, bottom gene = worst ranked).
(TIF)

S8 Fig MEVA outperforms its component methods, non-prioritized genes, and MAGMA in AoU. Heatmap displaying method performance across multiple validation criteria for genes significant in MEVA, the component methods, non-prioritized genes, and MAGMA. Cells are color-coded by rank (red = best, blue = worst) and annotated with either p-values (GWAS Colocalization, Gold Standard, and Mouse Phenotype Overlap) or z-scores (Gold Standard Broad and Direct Connectivity, Mouse Phenotype Broad Connectivity, and 1 + PubMed Co-Mentions). GO Term analyses are omitted as no method showed enrichment.
(TIF)

S9 Fig Replicated MEVA candidates show same directional effect. Lollipop plots of variants (AF < 50%) for NRIP1 (A-B; EA 70–100), TUBB1 (C-D; EA 30–70), and CALCOCO2 (E-F; EA 30–70) in UKB and AoU. Linear protein sequence is colored by the Evolutionary Trace and lollipops, representing single variant positions, are colored by EA. Lollipop height corresponds to the log10(Allele Count) in T2DM cases (top) and healthy controls (bottom).
(TIF)

S1 Table UKB Phenotypic Fields used to filter for T2DM Cohort. (XLSX)

S2 Table Gold Standard genes (n = 31) selected for high genetic association evidence and coding variant association evidence. (XLSX)

S3 Table P-Values and rankings for Gold Standards across each method. (XLSX)

S4 Table Gene Ontology Biological Process Enrichment for MEVA Candidates. Deprecated terms were removed from the Gene Ontology structure and were not assessed for term similarity.
(XLSX)

S5 Table Type 2 Diabetes Knowledge Portal GO Terms used as True Positives for Pathway Enrichment Comparison. (XLSX)

S6 Table T2DM-related (OMIM:125853) mammalian phenotypes curated by the Alliance for Genome Resources. (XLSX)

S7 Table MEVA candidates are enriched for mammalian phenotypes in mouse models. (XLSX)

S8 Table MEVA retains consistency and specificity in progressively smaller cohorts. Hypergeometric overlap between the top 100 genes in downsampled experiments and the top 100 full cohort genes, and the AUROC of T2DM gold standards (n = 31) across the full p-value rankings in down-sampled experiments.
(XLSX)

S9 Table The occurrence of UKB MEVA genes (n = 101) in each down-sampled experiment. (XLSX)

S10 Table AoU Phenotypic Fields used to filter for T2DM Cohort. (XLSX)

S11 Table AoU medication codes used in phenotypic filtering for T2DM cohort. (XLSX)

S12 Table 25 AoU nominally significant (p < 0.05) genes that overlap with the 101 UKB candidates. (XLSX)

S13 Table Gene-based odds ratios (OR) for 48 genes replicated in UKB and AoU. Odds ratios are stratified by EA scores (EA 70–100 approximating loss of function; EA 30–70 approximating gain of function) and Allele Frequency (AF < 50%; AF < 5%).
(XLSX)

S14 Table Pathway enrichment of MEVA gene clusters. Each cluster’s GO Biological-Process overlap with T2DKP terms is flagged (1 = overlap).
(XLSX)

S15 Table Functional prioritization of 177 MEVA candidates across measures of Robustness and relation to T2DM-associated genetics, pathways, and phenotypes. (XLSX)
