# Correction: Meta-evolutionary exome analysis identifies novel type 2 diabetes mellitus genes in the UK Biobank and All of Us

**DOI:** 10.1371/journal.pgen.1012265

**Published:** 2026-08-03

**Authors:** The PLOS Genetics Staff

## Notice of Republication

This article was republished on July 17, 2026, to correct an error in the article title. The words “All of Us” are miscapitalized in the article title. The correct title is: Meta-evolutionary exome analysis identifies novel type 2 diabetes mellitus genes in the UK Biobank and All of Us. The correct citation is: Wilhelm K, Asmussen J, Lee K, Samieinasab M, Asante E, Kimmel M, et al. (2025) Meta-evolutionary exome analysis identifies novel type 2 diabetes mellitus genes in the UK Biobank and All of Us. PLoS Genet 21(9): e1011889. https://doi.org/10.1371/journal.pgen.1011889. The publisher apologizes for the error. Please download this article again to view the correct version. The originally published, uncorrected article and the republished, corrected articles are provided here for reference.

## Supporting information

S1 File Originally published, uncorrected article. (PDF)

S2 File Republished, corrected article. (PDF)
