# Supplementary figures and images for: Meta-evolutionary exome analysis identifies novel type 2 diabetes mellitus genes in the UK Biobank and All of Us
Source: PLoS Genet. 2025 Sep 30;21(9):e1011889. doi: 10.1371/journal.pgen.1011889 (PMC12510657; doi:10.1371/journal.pgen.1011889)

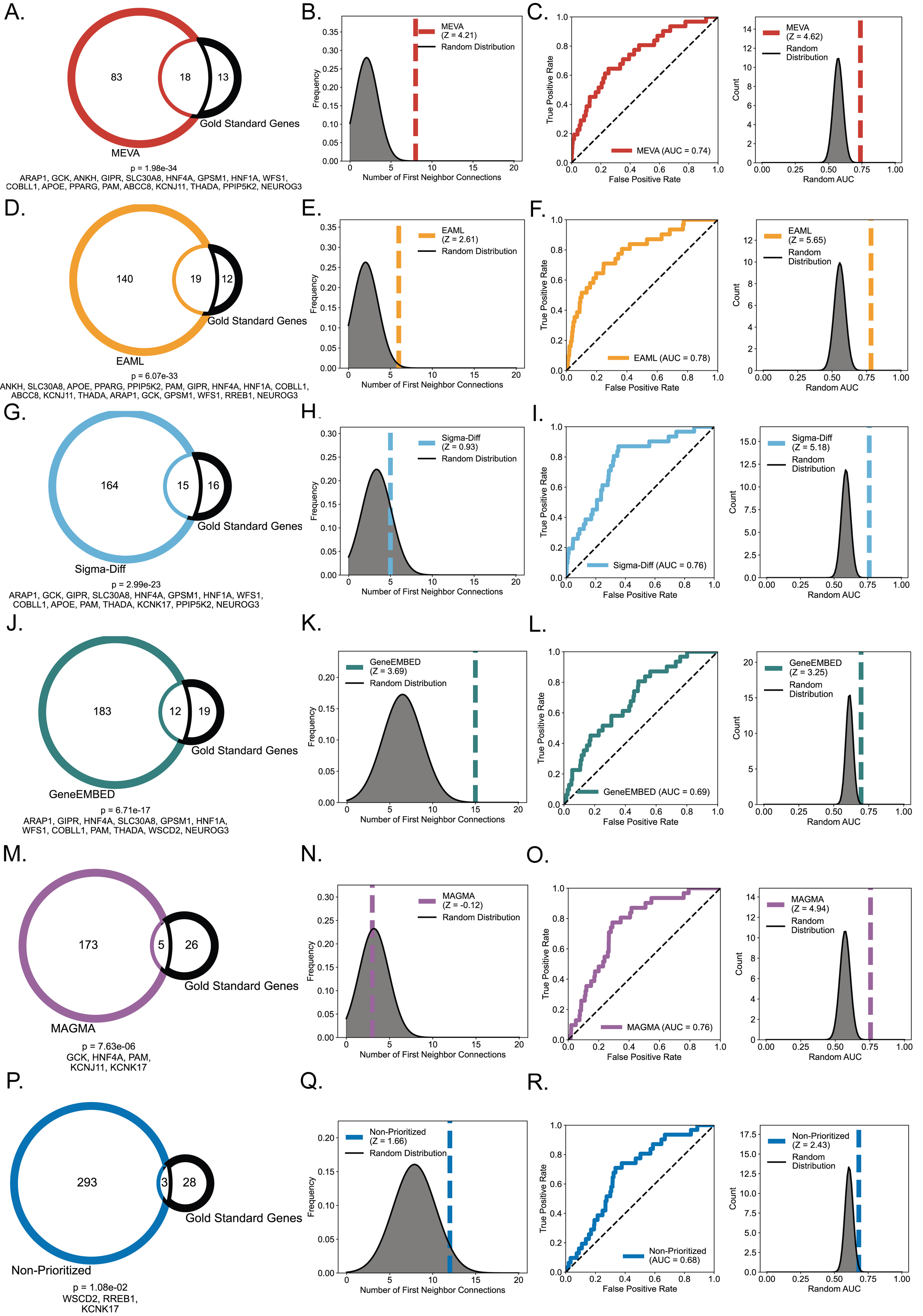

Supplement: S1 Fig — Panels A, D, G, J, M, P: Venn diagram showing overlap between each method’s gene list and the gold standards (hypergeometric test). Panels B, E, H, K, N, Q: First-neighbor connectivity of each method’s gene list to gold standards in STRING v12 (edge weight > 0.9). Significance determined by z-test versus 100 random, degree-matched sets. Panels C, F, I, L, O, R: Broad connectivity via network diffusion from each method’s gene list (excluding overlaps) to gold standards in STRING v12 (edge weight > 0). AUROC was calculated by ranking receiving nodes, with significance determined by z-test against AUROC’s of 100 random, degree-matched receiving nodes. (TIF) [file pgen.1011889.s001.tif]

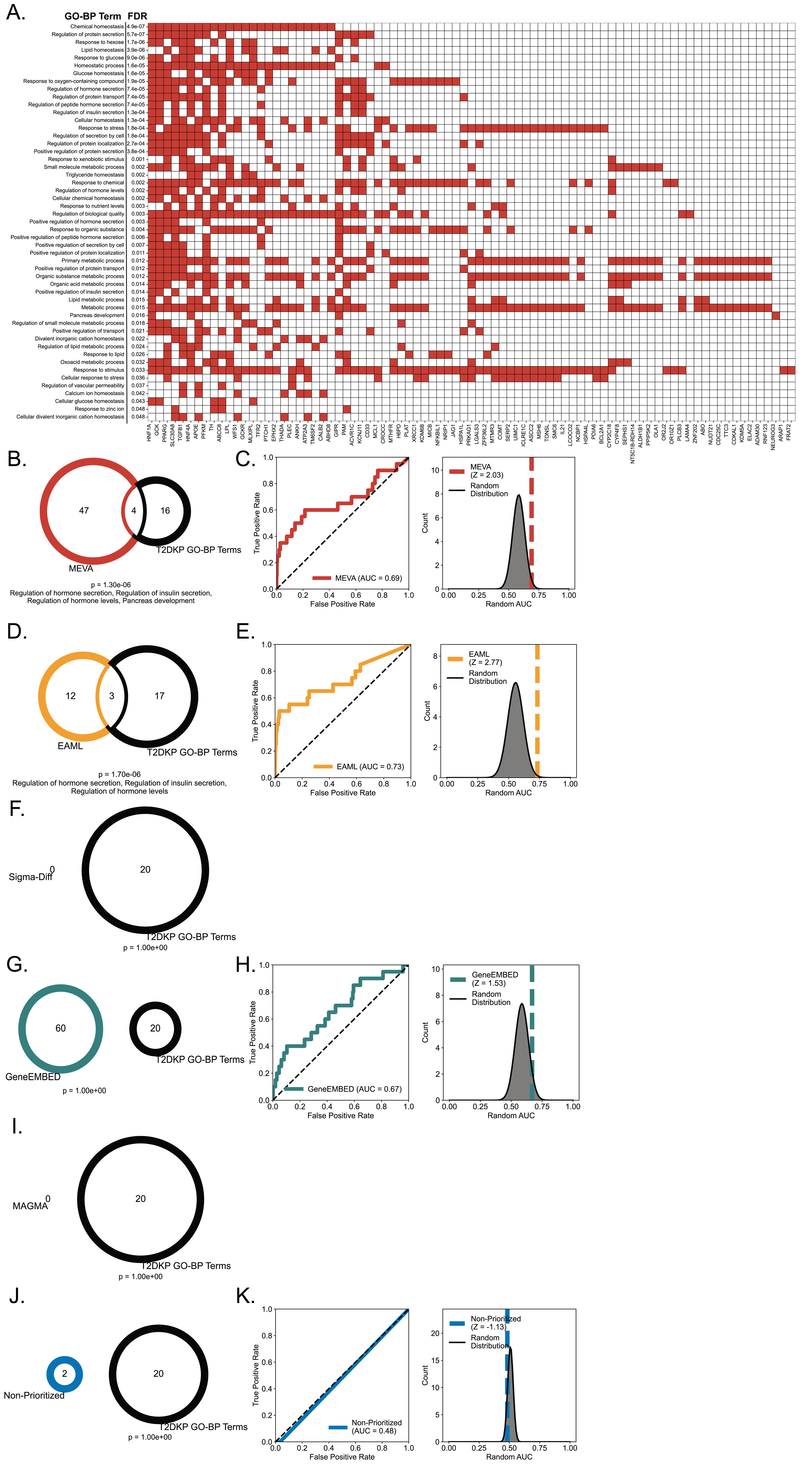

Supplement: S2 Fig — Panel A: Heatmap of MEVA-prioritized genes versus enriched GO-BP terms (FDR < 0.05), sorted by FDR. Red indicates gene membership in each term. Panels B, D, F, G, I, J: Venn diagrams of overlap between each method’s enriched GO-BP terms (FDR < 0.05) and the 20 T2DKP GO-BP terms (hypergeometric test). Panels C, E, H, K: Broad connectivity via network diffusion from each method’s enriched GO-BP terms (excluding overlaps) to the 20 T2DKP terms with the Gene Ontology structure. AUROC was calculated by ranking receiving terms, with significance determined by z-test against AUROC’s of 100 random, degree-matched receiving GO-BP terms. (Note: Sigma-Diff and MAGMA had no enriched terms and were not included in connectivity analyses). (TIF) [file pgen.1011889.s002.tif]

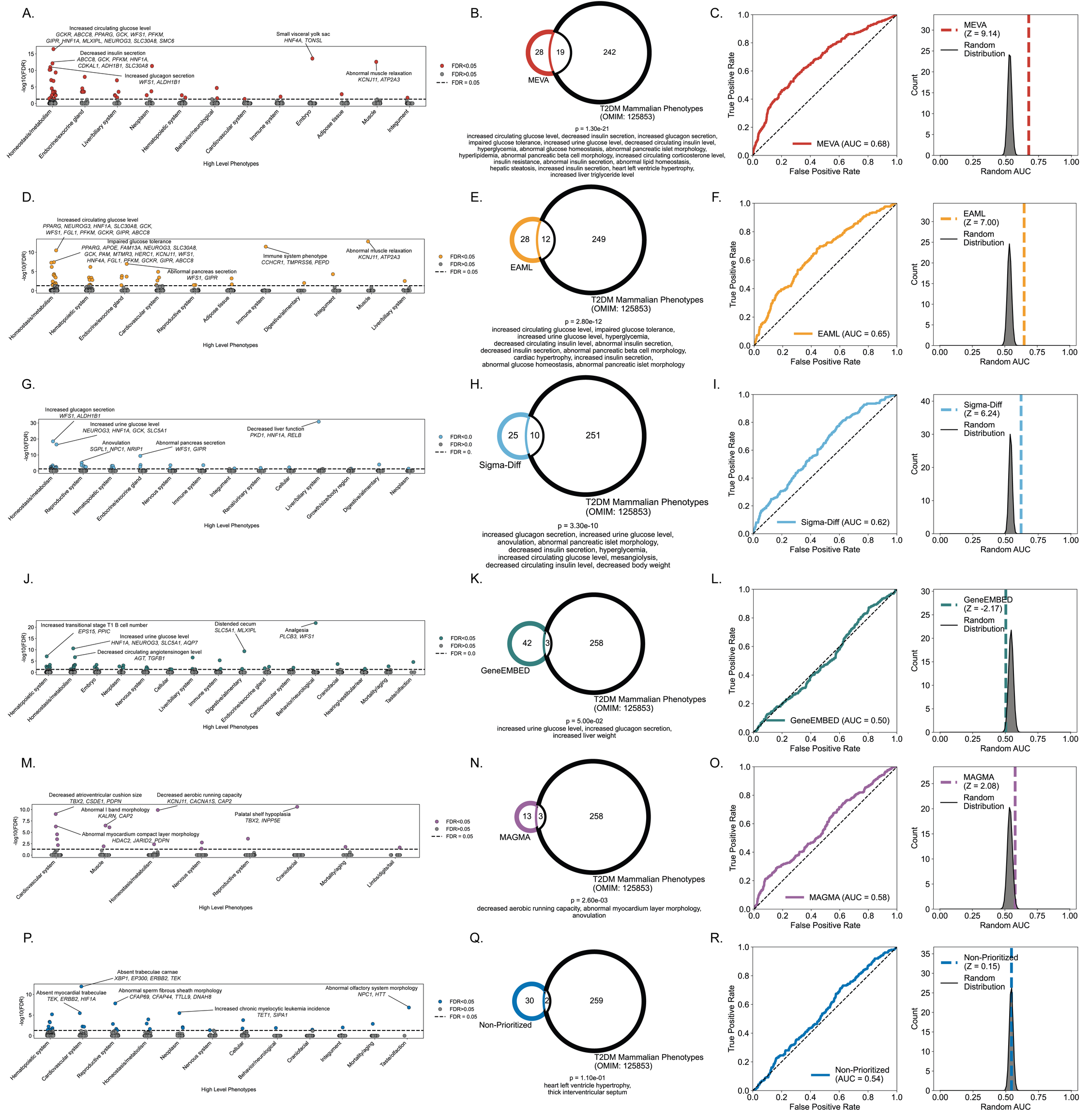

Supplement: S3 Fig — Panels A, D, G, J, M, P: Enrichment of mouse phenotypes for each method’s gene list. Each point represents a phenotypes -log10(FDR) (y-axis); colored if FDR < 0.05. Phenotypes are grouped into high-level categories (x-axis) and sorted by the number of significant phenotypes. The top five phenotypes are annotated with their label and associated genes. Panels B, E, H, K, N, Q: Venn diagrams between each method’s enriched phenotypes and the 261 T2DM-annotated mammalian phenotypes (hypergeometric test). Panels C, F, I, L, O, R: Broad connectivity via network diffusion from each method’s enriched phenotypes (excluding overlaps) to the 261 T2DM phenotypes in the mammalian phenotype ontology. AUROC was calculated by ranking receiving phenotypes, with significance assessed by z-test against AUROC distributions from 100 random, degree-matched phenotype sets. (TIF) [file pgen.1011889.s003.tif]

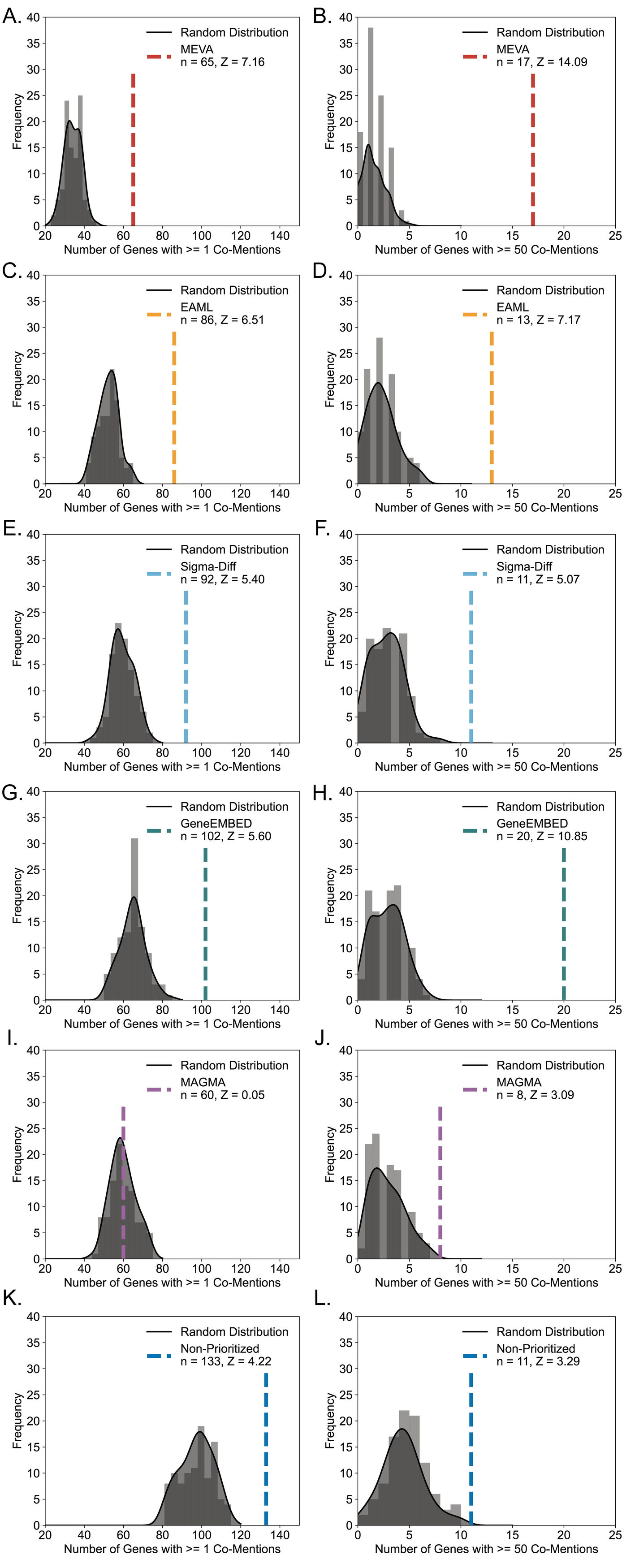

Supplement: S4 Fig — For each method’s gene list, we counted how many genes had ≥ 1 co-mention (Panels A, C, E, G, I, K) or ≥50 co-mentions (Panels B, D, F, H, J, L) with “Type 2 Diabetes”. The observed counts are shown as colored dotted lines against a null distribution (gray) generated from 100 random gene sets. Significance was assessed by z-test comparing the observed count to the random distributions. (TIF) [file pgen.1011889.s004.tif]

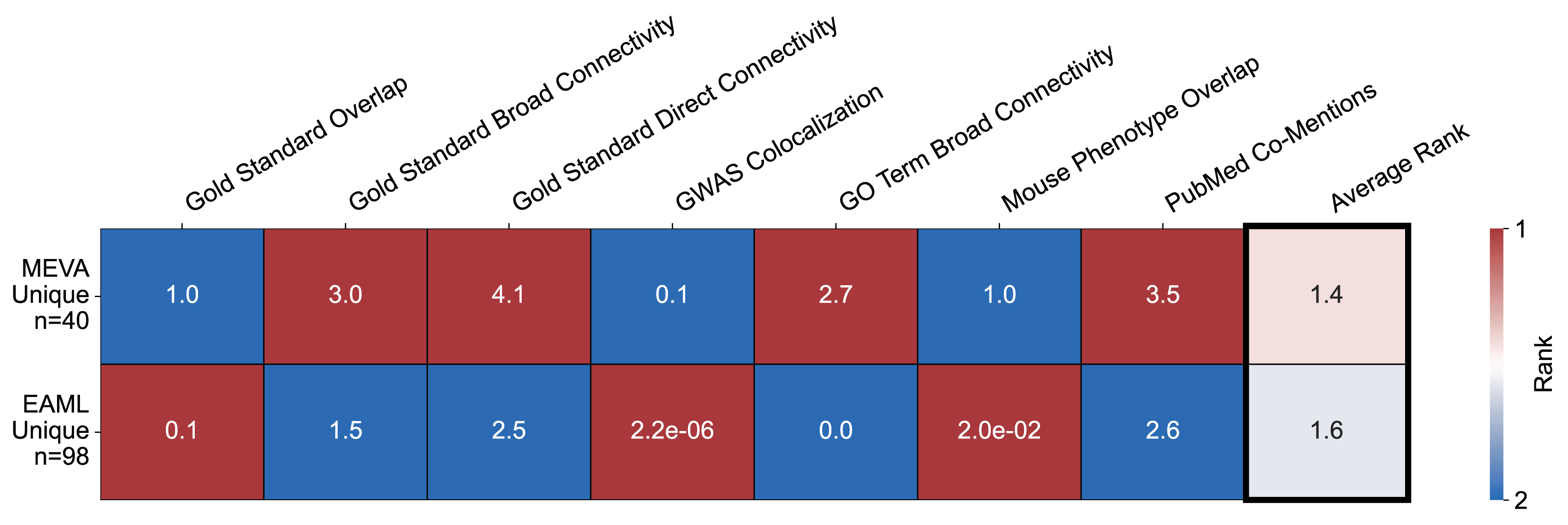

Supplement: S5 Fig — Heatmap displaying method performance across multiple validation criteria for genes unique to MEVA and EAML. Cells are color-coded by rank (red = best, blue = worst) and annotated with either p-values (GWAS Colocalization, Mouse Phenotype Overlap) or z-scores (Gold Standard Broad and Direct Connectivity, GO Term and Mouse Phenotype Connectivity, and 1 + PubMed Co-Mentions). Mouse phenotype connectivity and GO term overlap lacked enrichment in either set and were omitted. (TIF) [file pgen.1011889.s005.tif]

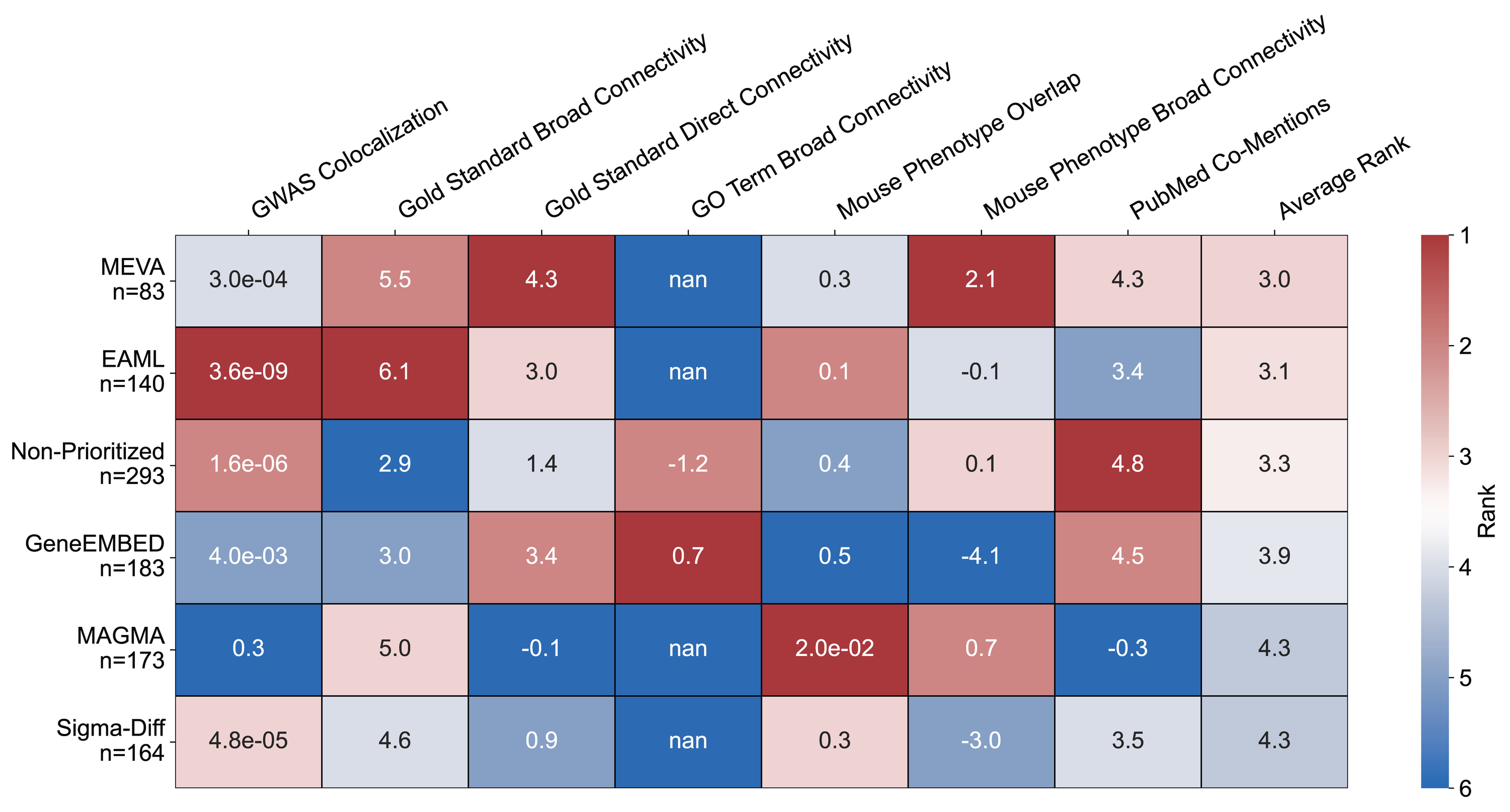

Supplement: S6 Fig — Heatmap displaying method performance across multiple validation criteria for genes significant in MEVA, the component methods, non-prioritized genes, and MAGMA. Cells are color-coded by rank (red = best, blue = worst) and annotated with either p-values (GWAS Colocalization, Mouse Phenotype Overlap) or z-scores (Gold Standard Broad and Direct Connectivity, GO Term and Mouse Phenotype Connectivity, and 1 + PubMed Co-Mentions). Gold standard overlap was removed due to the removal of all gold standards in each method. GO term overlap was removed since no methods overlapped with T2DKP terms. GO term broad connectivity nan values represent no enriched terms to test. (TIF) [file pgen.1011889.s006.tif]

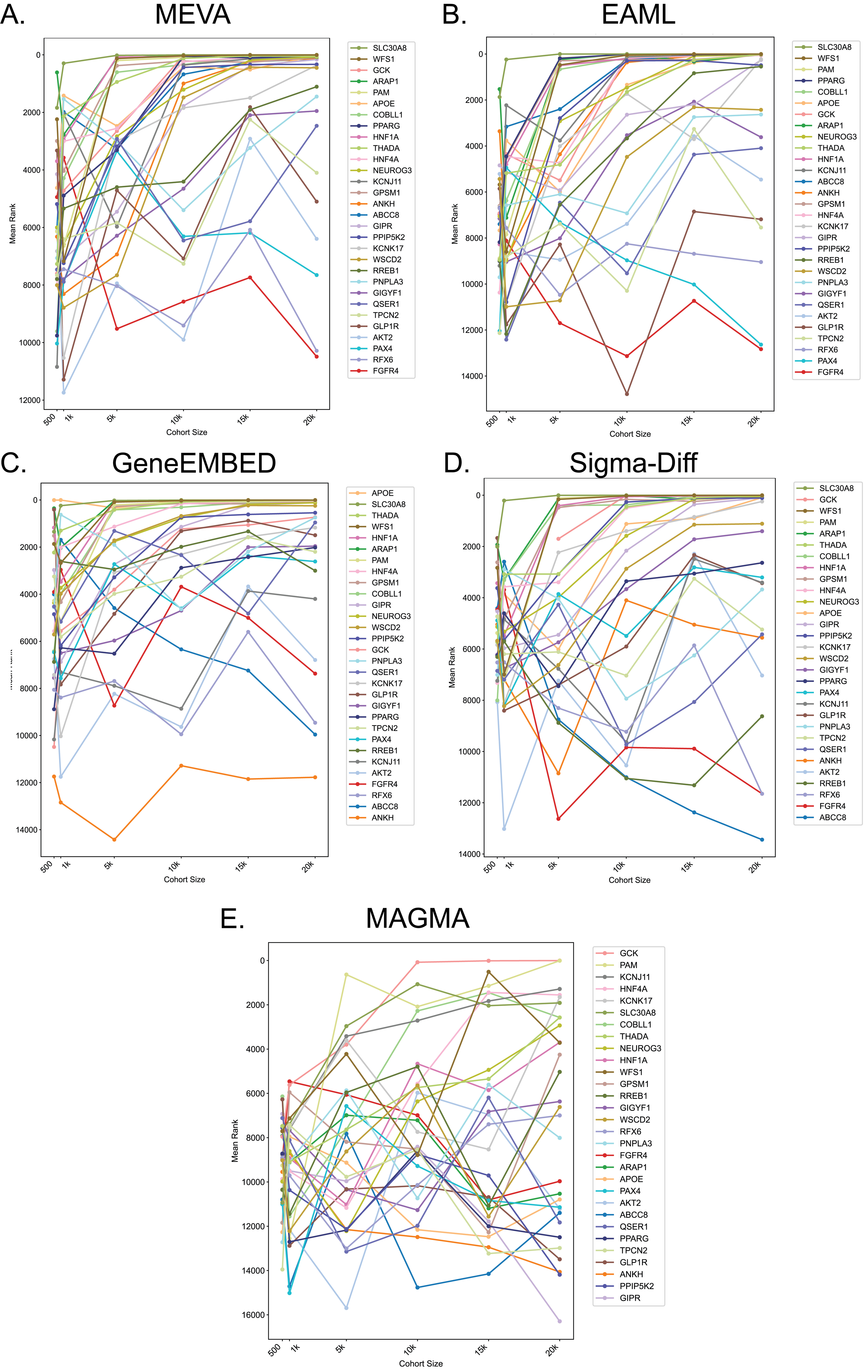

Supplement: S7 Fig — Average rankings (1 = most significant) of 31 T2DM gold standards across progressively smaller cohorts. Genes are color coded and sorted in each plot based on the mean rank in the 20k v. 20k experiments (top gene = best ranked, bottom gene = worst ranked). (TIF) [file pgen.1011889.s007.tif]

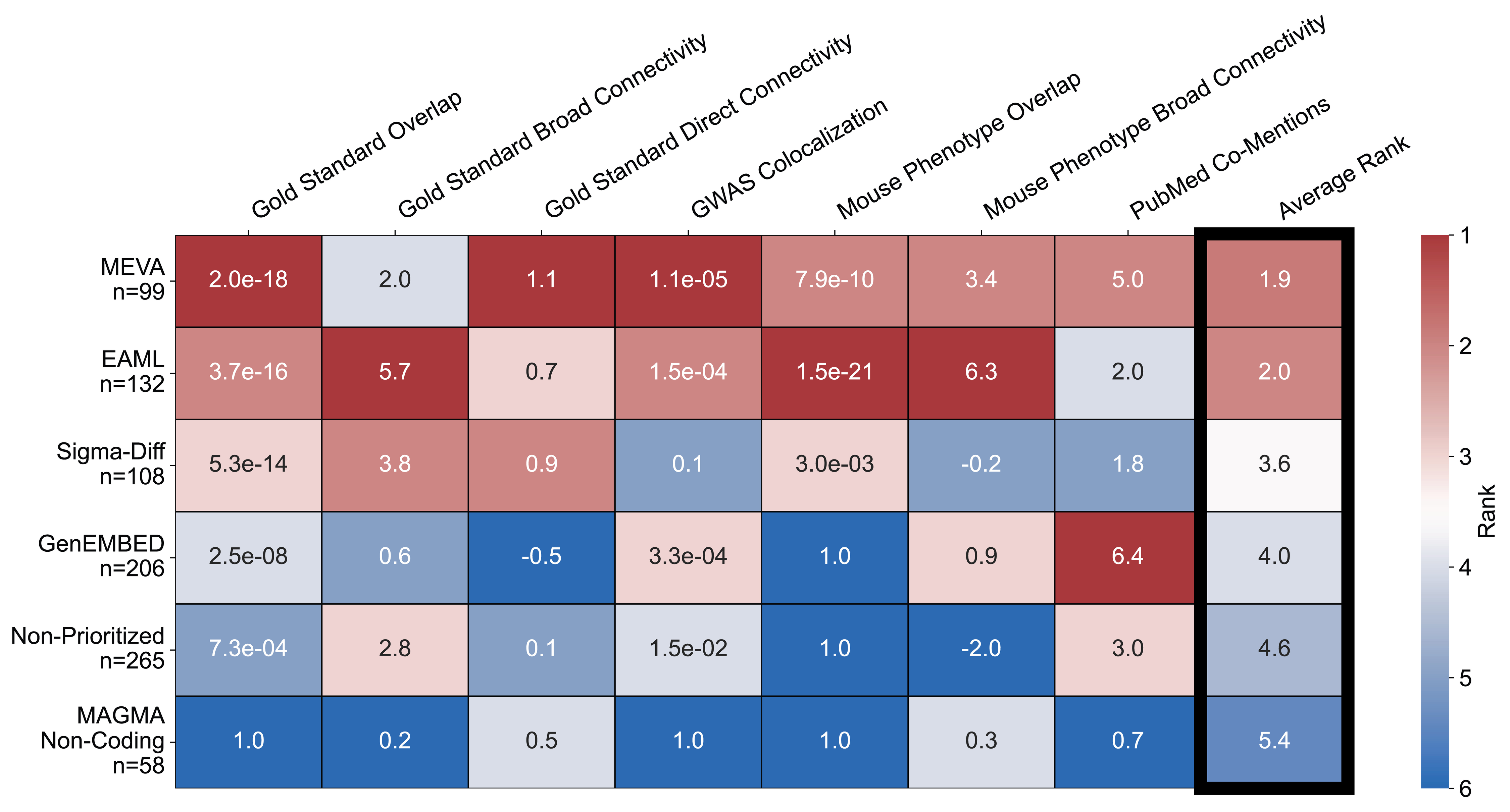

Supplement: S8 Fig — Heatmap displaying method performance across multiple validation criteria for genes significant in MEVA, the component methods, non-prioritized genes, and MAGMA. Cells are color-coded by rank (red = best, blue = worst) and annotated with either p-values (GWAS Colocalization, Gold Standard, and Mouse Phenotype Overlap) or z-scores (Gold Standard Broad and Direct Connectivity, Mouse Phenotype Broad Connectivity, and 1 + PubMed Co-Mentions). GO Term analyses are omitted as no method showed enrichment. (TIF) [file pgen.1011889.s008.tif]

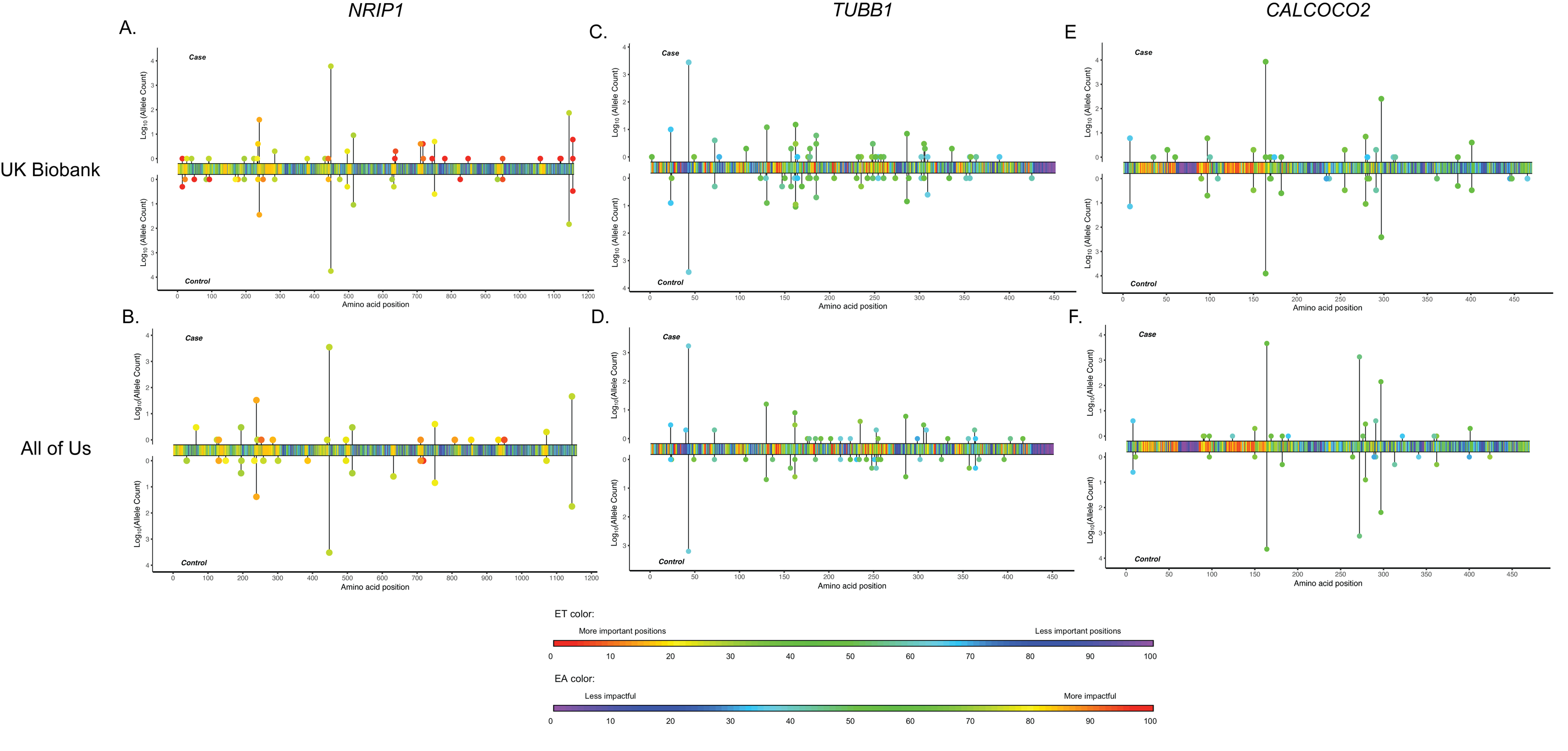

Supplement: S9 Fig — Lollipop plots of variants (AF < 50%) for NRIP1 (A-B; EA 70–100), TUBB1 (C-D; EA 30–70), and CALCOCO2 (E-F; EA 30–70) in UKB and AoU. Linear protein sequence is colored by the Evolutionary Trace and lollipops, representing single variant positions, are colored by EA. Lollipop height corresponds to the log10(Allele Count) in T2DM cases (top) and healthy controls (bottom). (TIF) [file pgen.1011889.s009.tif]
